# Genome-wide association analysis identifies genetic loci associated with resistance to multiple antimalarials in *Plasmodium falciparum* from China-Myanmar border

**DOI:** 10.1038/srep33891

**Published:** 2016-10-03

**Authors:** Zenglei Wang, Mynthia Cabrera, Jingyun Yang, Lili Yuan, Bhavna Gupta, Xiaoying Liang, Karen Kemirembe, Sony Shrestha, Awtum Brashear, Xiaolian Li, Stephen F. Porcella, Jun Miao, Zhaoqing Yang, Xin-zhuan Su, Liwang Cui

**Affiliations:** 1 Department of Entomology, Pennsylvania State University, University Park, PA 16802, USA; 2 Rush Alzheimer’s Disease Center, Rush University Medical Center, Chicago, IL, 60612, USA; 3 Department of Neurological Sciences, Rush University Medical Center, Chicago, IL, 60612, USA; 4 Department of Pathogen Biology and Immunology, Kunming Medical University, Kunming, Yunnan Province 650500, China; 5 Genomics Unit, Research Technologies Section (RTS), Rocky Mountain Laboratories (RML), NIAID, National Institutes of Health, 904 South 4th Street, Hamilton, MT 59840, USA; 6 Laboratory of Malaria and Vector Research, NIAID, National Institutes of Health, 9000 Rockville Pike, Bethesda, Maryland 20892, USA

## Abstract

Drug resistance has emerged as one of the greatest challenges facing malaria control. The recent emergence of resistance to artemisinin (ART) and its partner drugs in ART-based combination therapies (ACT) is threatening the efficacy of this front-line regimen for treating *Plasmodium falciparum* parasites. Thus, an understanding of the molecular mechanisms that underlie the resistance to ART and the partner drugs has become a high priority for resistance containment and malaria management. Using genome-wide association studies, we investigated the associations of genome-wide single nucleotide polymorphisms with *in vitro* sensitivities to 10 commonly used antimalarial drugs in 94 *P. falciparum* isolates from the China-Myanmar border area, a region with the longest history of ART usage. We identified several loci associated with various drugs, including those containing *pfcrt* and *pfdhfr*. Of particular interest is a locus on chromosome 10 containing the *autophagy-related protein 18 (ATG18*) associated with decreased sensitivities to dihydroartemisinin, artemether and piperaquine – an ACT partner drug in this area. ATG18 is a phosphatidylinositol-3-phosphate binding protein essential for autophagy and recently identified as a potential ART target. Further investigations on the ATG18 and genes at the chromosome 10 locus may provide an important lead for a connection between ART resistance and autophagy.

Malaria parasites put ~3.2 billion people at risk and claim over 1,000 lives in the world every day^1^. Of the four major human malaria parasites, *Plasmodium falciparum* is the most virulent causing the majority of global malaria-related mortality. Because of the widespread resistance in this parasite to multiple antimalarial drugs including chloroquine (CQ), sulfadoxine-pyrimethamine (SP), mefloquine (MQ) and quinine (QN), artemisinin (ART)-based combination therapies (ACTs) have become the frontline treatment of falciparum malaria since 2005. To our dismay, clinical resistance to ARTs also emerged recently in *P. falciparum*. ART resistance manifested as delayed parasite clearance (DPC) has been widely reported in Southeast Asia^2^,^3^,^4^. More worrisome, the recent identification of clinical resistance to dihydroartemisinin (DHA)/piperaquine (PPQ) in Cambodia indicates that parasites are evolving resistance to the combination drugs^5^,^6^. Therefore, understanding the genetic basis underlying *P. falciparum* resistance remains a high priority in order to curb its emergence elsewhere and reduce global spread of this human pathogen.

The DPC phenotype, defined as half-life of the parasite clearance >5 h, is postulated to be the result of dormant ring-stage parasites, which are able to endure high concentrations of ARTs for a short period of time^7^. The DPC phenotype therefore does not correspond to the traditional definition of drug resistance of increased half maximum inhibitory concentration (IC_50_) values within *in vitro* parasite proliferation assays. A ring-stage survival assay (RSA) that measures the survival rate of 0–3 h ring-stage parasites after 6 h exposure to 700 nM for of DHA was developed^8^. The RSA results generally have a good correlation with *in vivo* parasite clearance half-lives^9^,^10^. Given the significant role of ACTs in contemporary malaria control, the genetic basis of ART resistance has been actively pursued, leading to the identification of a locus on chromosome 13 and a gene with kelch-propeller (K13) within the locus that are linked to DPC^11^,^12^,^13^. Several mutations within the K13 propeller domain have been genetically validated as mediating ART resistance^14^,^15^. To explore the underlying molecular mechanisms, transcriptome analysis showed that clinically resistant parasites have an altered developmental cycle with a decelerated ring stage and an upregulation of unfolded protein response pathway^16^. Recently, phosphatidylinositol-3-kinase (PI3K) signaling was proposed as a target of ART, and K13 mutations were found to increase PI3K phosphorylation level^17^. In this case, K13 is believed to be responsible for the recruitment of an ubiquitin ligase to PI3K, leading to its ubiquitination and degradation. Several K13 mutations reduced the interaction with PI3K, which led to reduced PI3K ubiquitination and increased PI3K activity. The link of ART resistance to protein ubiquitination also emerged in other studies. ART drugs have been shown to affect the parasite’s cell stress response involving the ubiquitin/proteasome system, which is enhanced by certain K13 mutations^18^. In ART-resistant parasites (with K13 mutations), overall protein ubiquitination was reduced. Studies using ART activity-based protein profiling probes and covalent binding of ARTs to multiple parasite targets suggest that ART resistance may involve additional genes^19^,^20^. Therefore, further studies are needed to elucidate molecular mechanisms underlying the complex trait of ART resistance. Equally important are the identification of molecular markers associated with resistance to other ACT partner drugs.

The Greater Mekong Subregion (GMS) of Southeast Asia is an epicenter of antimalarial drug resistance, where parasites resistant to CQ and pyrimethamine have emerged initially and spread to Africa. As feared, ART resistance also emerged in the same location. Therefore, drug resistance management remains an important challenge in malaria control in the GMS. In this region, Myanmar has the heaviest malaria burden^21^. Moreover, Myanmar shares extensive international borders with Thailand and China in the east and with Bangladesh and India in the west, making it an important bridge for spreading resistance to South Asia and Africa. In southern Myanmar, markedly delayed clearance after ART treatment has been detected in approximately 1/3 of *P. falciparum* infections^22^. Similarly, increases in day-3 parasite positive cases to well over 10% after ACT treatment, a substitute of ART resistance, and prevalence of K13 mutations in the eastern borders with China and Thailand suggest emergence and spread of ART resistance in this region^10^,^23^,^24^. In addition, DPC after ART treatment was also detected in Yunnan province at the China-Myanmar border^25^, where ARTs had the longest history of deployment, highlighting the significance of increased regional efforts in drug resistance surveillance. Furthermore, the China-Myanmar border area has had quite different antimalarial drug use history from other GMS countries. CQ, SP, and QN have been widely used in the past, while CQ is still the frontline treatment for the sympatric *P. vivax* malaria. From the late 1970 s, ART derivatives and pyronaridine (PND) were recommended as the second-line drugs in place when CQ and PPQ resistance was high^26^. In the mid-1980 s, artemether (AM) was widely used to treat falciparum malaria. In the early 1990 s, artesunate (AS) and DHA were introduced. Beginning in 2005, ACTs [mostly DHA/PPQ as well as AM/lumefantrine (LMF)] began to replace ART monotherapies^27^. This distinctive antimalarial drug history may account for the quite different antimalarial drug sensitivity profiles and genetic structures of the parasites from this region^28^,^29^,^30^,^31^. Therefore, genetic studies of parasites from this region may reveal new mechanisms of resistance to ART derivatives and other antimalarials.

In an effort to survey drug resistance in other sentinel sites in the GMS and identify additional genetic mechanisms of ART resistance, we analyzed *P. falciparum* clinical isolates from a longitudinal collection of clinical parasite isolates originating from the China-Myanmar border area of the GMS. By assessing *in vitro* parasite sensitivities to multiple antimalarial drugs and performing genome-wide genotyping, we aimed to identify genomic regions that are associated with drug resistant phenotypes. In addition to *pfcrt* and *pfdhfr*, which are known to confer resistances to CQ and SP, respectively, we also identified several new loci associated with different drugs, including a locus on chromosome 10 that is significantly associated with parasite responses to ART and PPQ.

## Results

### *In vitro* sensitivities of field isolates to antimalarial drugs

**Figure 1:**
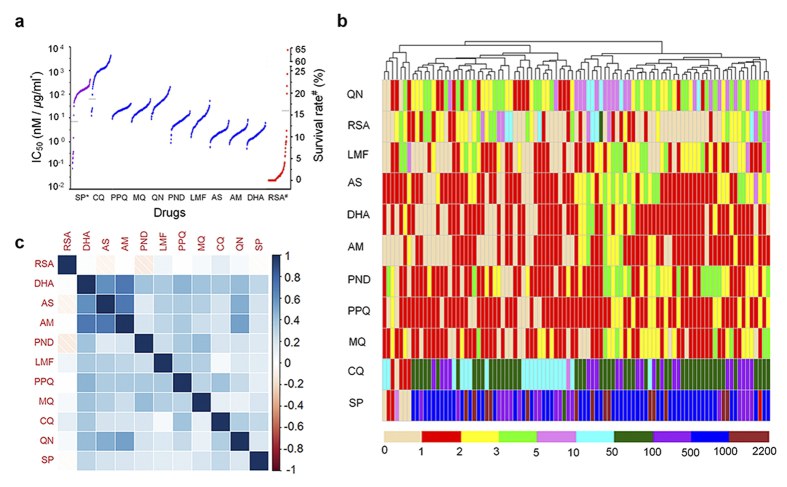
*In vitro* parasite sensitivities to ten antimalarial drugs. (**a**) IC_50_ values to 10 different drugs and ring-stage parasite survival rates measure by RSA were sorted from the lowest to the highest values. IC_50_ values to SP are shown in *μ*g/ml, and RSA values are in percentage. Large discontinuous gaps were present in IC_50_ values to SP and CQ, and ring-stage survival rates to DHA. Dashed line indicates the separation. (**b**) Fold change of drug sensitivities of field isolates in comparison with those of 3D7. Each column represented the sensitivities of a field isolate to 10 drugs. Hierarchical clustering of parasite isolates was analyzed using “ward” method in the R package *stats*. The degree of fold change is colour-coded. (**c**) Correlations between drug sensitivities of parasite isolates to ten antimalarial drugs. The correlations between drug sensitivities were analyzed by Spearman’s test. The degree of correlation between sensitivities of two drugs is color-coded. Drug Abbreviations: chloroquine (CQ); sulfadoxine-pyrimethamine (SP); ring-survival rates from RSA; dihydroartemisinin (DHA); artemether (AM); piperaquine (PPQ); quinine (QN); lumefantrine (LMF); and pyronaridine (PND).

We evaluated *in vitro* sensitivities of 94 culture-adapted parasite isolates and the laboratory clone 3D7 to 10 commonly used antimalarial drugs using a SYBR Green I assay and RSA (Fig. 1a and Supplementary Table S1). With the standard drug assay that measures parasite proliferation, parasite isolates were found to be relatively sensitive to ART family drugs with a median IC_50_ value of 1.9 nM to DHA, and 2.5 nM to AS and AM, although the ranges were wide. In contrast, with the RSA which measures the survival rate of ring-stage parasites after short exposure to a bolus DHA treatment, the median survival rate of these clinical isolates was 0.7%, and the range was also very broad (0–64.03%). CQ resistance prevailed in these clinical isolates, with only five sensitive isolates present in the study samples (Fig. 1a,b). Similarly, most parasites were also very resistant to SP with a median IC_50_ value of 139.7 μg/ml and a broad range with ~4,000-fold difference. Though cutoffs to define resistance to CQ and SP were not defined here, the resistant and sensitive isolates were clearly separated by large gaps in their *in vitro* IC_50_s (Fig. 1a). Likewise, parasites also had relatively wide ranges of IC_50_ values to LMF, PND and QN, with >20-fold differences in IC_50_ between the most and the least susceptible isolates. In comparison, parasites showed fairly narrow ranges of IC_50_ to PPQ and MQ (<10-fold difference). A comparison between the clinical isolates and 3D7 revealed statistically significant decreases in sensitivity to all 10 drugs tested in the field isolates (*P* < 0.0001, Student’s *t*-test, Table S1). A significantly higher median ring survival rate was also observed in clinical isolates compared to 3D7 (*P* = 0.0003). Hierarchical clustering analysis on the fold changes of drug sensitivities from field isolates as compared with those of 3D7 clearly illustrated multidrug resistance phenotypes in many isolates (Fig. 1b). Yet, stratification of the IC_50_ data by year did not reveal significant differences between the years. Pairwise comparison between the 10 drugs by Spearman’s rho analysis revealed highly significant, positive, correlations among the IC_50_s of DHA, AS and AM. DHA and PPQ also showed significant positive correlations with all other drugs. Significant positive correlation was also noticed between the following pairs of drugs: PND and MQ, LMF and MQ, CQ and QN, and CQ and SP. However, there was little correlation between RSA results and the IC_50_s of the tested drugs (Fig. 1c), indicating that these two assays measure two different drug response phenotypes.

### Single nucleotide polymorphism (SNP) array genotypes

We performed genome-wide genotyping of the parasite isolates utilizing a high-density Affymetrix SNP array^32^. The SNP array detected a total of 17,582 (100%) high-confidence SNPs at a minimum of 90% call rate in all samples, giving an approximate density of one SNP per kb. Low-frequency SNPs [minor allele frequency (MAF) <0.02)] predominated at 40.4% in the samples (Supplementary Fig. S1). For quality control, we first removed 1,506 (8.6%) SNPs within the multigene families *var*, *rifin* and *stevor* to avoid artifacts from duplicated sequences. Furthermore, 779 SNPs (1.4%) with more than 10% missing genotypes were also excluded. Missing data in the remaining 15,297 SNPs were imputed by Beagle^33^, which is a linkage disequilibrium (LD)-based imputation program that has the ability to accurately impute missing genotypes and improve the accuracy of association analyses in *P. falciparum*^34^. This was further filtered to remove multi-allelic and low-frequency SNPs with a MAF of <2%, resulting in a final dataset consisting of 8,564 SNPs. These SNPs are unevenly distributed across the genome with an average distance of ~4.2 kb between adjacent SNPs (Supplementary Fig. S2) with the MAF ranging from 2.1% to 49.5%.

### Population structure and LD

**Figure 2:**
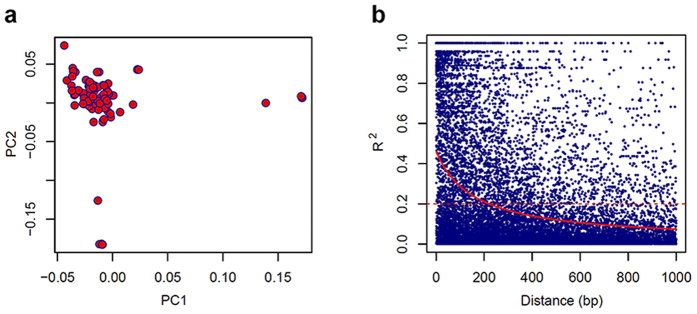
Population structure and linkage disequilibrium in the parasite population. (**a**) Population partitions identified by PCA, showing two minor outlier groups. (**b**) Plot of LD measured as squared correlation of allele frequencies (R^2^) against physical map distance (bp) between linked locus pairs in the entire population. The red solid line is the nonlinear regression trend line of R^2^ vs. physical map distance, and dashed red line indicates R^2^ = 0.2.

To determine whether the study population is genetically structured, we performed principal component analysis (PCA, Fig. 2a). This analysis revealed partitioning of the parasites into one major and two minor clusters, indicating that most of the isolates had a similar genetic background. The genome-wide average LD level in this population, as expected, decayed rapidly within a few hundred base pairs and reached a baseline level within 1000 bp (Fig. 2b). Compared to other populations, LD decayed to R^2^ = 0.2 within approximately 220 bp in this parasite population. This rate was slower than that in parasites from Cambodia and Thailand, which were reported to be within 187 and 162 bp^34^, respectively, suggesting a lower level of outcrossing and less effective recombination. This result is also consistent with the lower estimated multiplicity of infection (mean, 1.41) in the parasite population from the China-Myanmar border^35^ than that from the Thailand-Myanmar border (mean, 2.15)^36^ analyzed using the polymorphic marker *merozoite surface protein 1 (msp1*). However, this estimated LD decay rate was only an approximation given the average SNP resolution of 4.2 kb, while a more accurate calculation requires higher-density SNPs from whole genome sequencing. In addition, despite the overall fast decay in LD, there were a number of SNP pairs with much higher R^2^ values (R^2^ > 0.3), passing the average distance of 4.2 kb (Supplementary Fig. S3).

### Genetic loci linked to parasite responses to drugs

**Figure 3:**
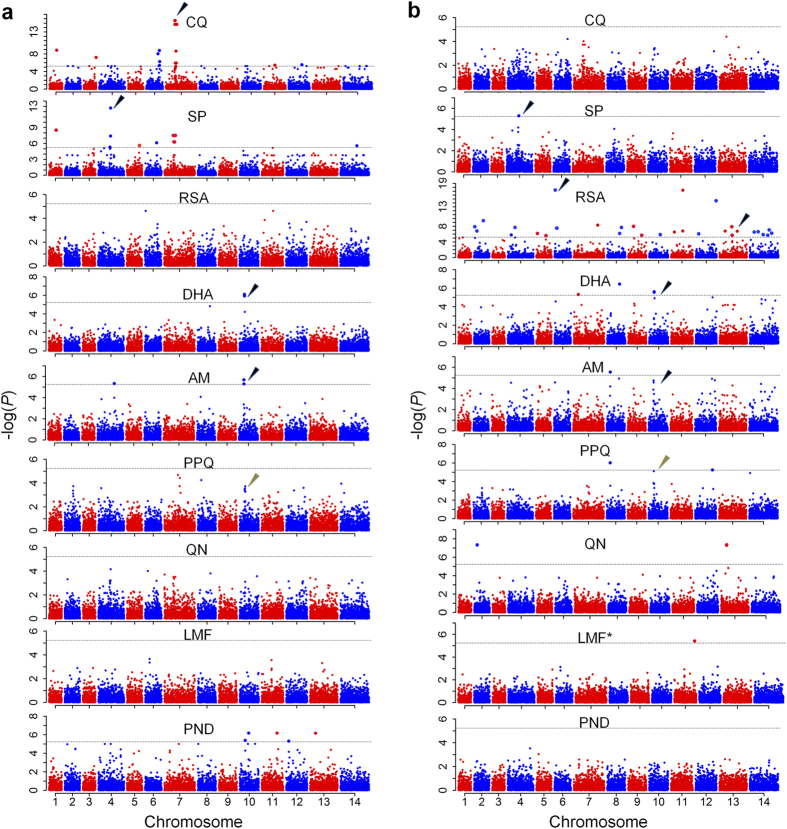
Manhattan plots showing the significance of SNP association in the GWAS. Values of –log(*P*) for 10 drugs were plotted against chromosomal positions of SNPs. Each point represents 1 of 8572 SNPs with MAF >0.02 in a set of 94 isolates. The dashed horizontal line indicates the significance threshold of a *P* value of 5.83 × 10^−6^ after Bonferroni correction. (**a**) Analysis with log-transformed phenotypic data; (**b**) Analysis with original phenotypic data. Plots are from GEMMA, except LMF* from PLINK. The arrowheads indicate interesting SNPs. Drug Abbreviations: chloroquine (CQ); sulfadoxine-pyrimethamine (SP); ring-survival rates from RSA (DHA^RSA^); dihydroartemisinin (DHA); artemether (AM); piperaquine (PPQ); quinine (QN); lumefantrine (LMF); and pyronaridine (PND).

To detect genes associated with altered susceptibilities to the 10 antimalarial drugs, we conducted a genome-wide association study (GWAS) using the IC_50_ values as continuous outcomes. To increase the robustness of the study, we used multiple programs, including genome-wide efficient mixed-model association (GEMMA)^37^, PLINK^38^, linear-regression and nonparametric regression in R, and warped linear mixed model (WarpedLMM)^39^. Whereas linear regression analysis assumes a Gaussian distribution of quantitative phenotypes and requires data normalization, a recent study suggested that data transformations are typically not needed^40^. We therefore exploited both original and normalized drug sensitivity data in our GWAS. The top three principal components were applied as covariates to reduce the influences of population stratification. Quantile-quantile (Q-Q) plots showed that the *λ* values approached 1 for all drugs, indicating effective correction of the population structure (Supplementary Fig. S4). A *P* value of 5.83 × 10^−6^ was used as the threshold after Bonferroni correction to assess the significance. As an internal validation of our GWAS, we first assessed associations of known molecular markers *pfcrt, pfdhfr* and *pfdhps* for resistance to CQ, pyrimethamine and sulfadoxine, respectively. Different GWAS programs showed different sensitivities in identifying resistance-associated SNPs. Moreover, exploitation of different phenotypic formats also yielded different results. GEMMA and PLINK with log-transformed phenotype data identified 17 and 46 significant SNPs for CQ, respectively, and the *pfcrt* locus was among the most significant association when normalized phenotype data were used (Fig. 3, Supplementary Fig. S5, and Supplementary Table S2). Flanking genes including *pfcg1*, *glp3*, *cg2*, *cg7*, *lysophospholipase* and a conserved gene were also identified as significantly associated with CQ resistance by GEMMA and/or PLINK. Consistently, strong LD (R^2^ > 0.3) was detected in a wide region (~177 kb) around *pfcrt* that harbors these genes (Table S3). Besides *pfcrt*, a region on chromosome 6 where a phospholipase, an amino acid transporter gene (PF3D7_0629500) and a SET domain protein are located was also positively associated with CQ resistance in the study population. Similarly, the most significant correlation between *pfdhfr* with SP resistance was detected, and a region of ~75 kb flanking the SP gene showed strong LD, highlighting the feasibility of the GWAS approach for identifying SNPs associated with altered drug sensitivities (Fig. 3, Supplementary Fig. S5 and Supplementary Tables S2 and S3). Interestingly, nine common SNPs were identified as significantly associated with resistance to both CQ and SP (Table S2), including one in *pfcrt*.

Given that clinical ART resistance is associated with an increased ring survival rate, we performed GWAS using both IC_50_ values and RSA results. Of particular interest, an autophagy-related protein on chromosome 10, *ATG18* (PF3D7_1012900), along with a neighbor gene *NLI interacting factor-like phosphatase* (PF3D7_1012700, NIF4) showed a strong association with increased IC_50_s to both DHA and AM in multiple analyses. The SNP in *pfatg18* corresponds to a T38N mutation and is present in 42.6% of the study population. Interestingly, this locus was approaching significance in association with decreased sensitivities to PPQ, a partner drug in the ACT mostly used at the China-Myanmar border (Fig. 3, Supplementary Fig. S5, Table 1, and Supplementary Table S2). It is noteworthy that a recent large-scale GWAS also detected the association between NIF4 with delayed parasite clearance half-life (PC*t*_1/2_) in patients after treatment with an ART derivative in other locations in Southeast Asia^41^, and ATG18 was recently identified as a potential ART target as it was found covalently bound to an ART analog^19^. In addition, strong LD correlation was observed in a very wide range spanning ~905 kb on chromosome 10, where a gene encoding *DNA polymerase delta catalytic subunit* (PF3D7_1017000) was reported to be associated with DPC in Southeast Asia^12^. In comparison, association with the original RSA result identified positive associations with genes on nearly all chromosomes, of which five were shared with the positives identified when the analysis was performed with the normalized RSA result. These analyses identified *DNA repair protein RAD5* (PF3D7_1343400), a gene within 6 kb of K13, as significantly associated with the RSA phenotype. Consistently, this gene was previously detected to be associated with DPC in Southeast Asia^12^. In addition, two genes, PF3D7_0602400 (*elongation factor G*) and PF3D7_0602500 (*geranylgeranyltransferase)*, were identified by several GWAS methods with the highest *P*-values.

For *in vitro* sensitivities to PPQ, QN, LMF and PND, significant associations were identified with a number of SNPs (Table 1 and Table S2). Of note, three SNPs showed association with PPQ resistance, although they were only detected by one method. Of these, the *GTPase-activating protein* on chromosome 8 (PF3D7_0804900) was the most significant. Seven SNPs were associated with IC_50_ values of QN, among which the PPM6 (PF3D7_1309200) protein on chromosome 13 and a conserved protein (PF3D7_0205800) on chromosome 2, which flanks a *DNA repair protein RAD2*, also were correlated with increased ring survival rates. This study identified five loci on chromosome 10 and 13 that were associated with sensitivities to PND. In contrast, only a *Plasmodium* exported protein (hyp11, PF3D7_1148800) was associated with reduced LMF sensitivity.

### Evidence of selection on drug resistance loci

**Figure 4:**
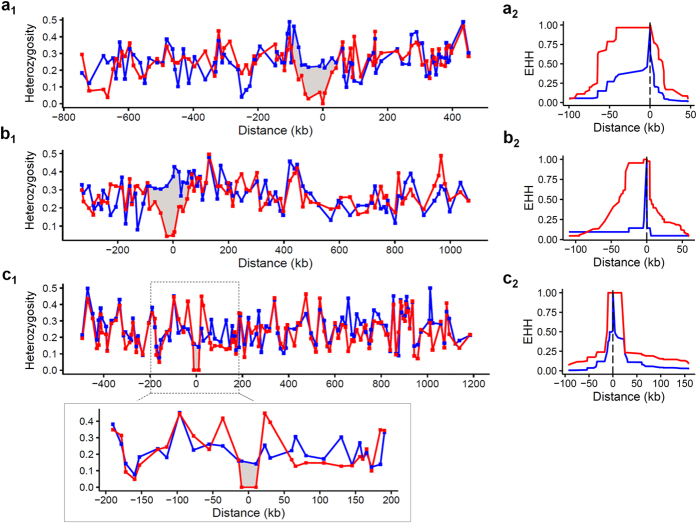
Selection around resistant loci. The SNP diversity on chromosome 4 (**a**_**1**_), 7 (**b**_**1**_) and 10 (**c**_**1**_) are shown by the measurement of average heterozygosity of each gene, centered at *pfdhfr*, *pfcrt* and *pfatg18*, respectively, and the rectangle subpanel of **c_1_** shows the magnified view of the *pfatg18* region. Red line represents SP- or CQ-resistant samples, as well as samples having the T38N mutation in *pfatg18*, and blue line stands for SP- or CQ-sensitive samples, or wild types in *pfatg18*. The disparity in diversity with grey shading likely reflects the selective sweep around resistant loci. EHH decays around *pfdhfr*, *pfcrt* and *pfatg18* are shown in **b_1_**, **b_2_** and **b_3_**, with S220A in *pfcrt*, C59R in *pfdhfr* and T38N in *pfatg18* as the focal SNPs. Red line indicates EHH decays in our samples, and blue line shows the decay in the reference line 3D7.

**Figure 5:**
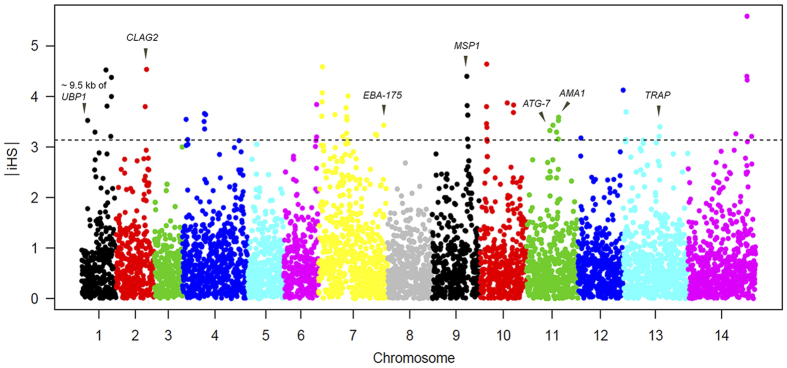
Plot of integrated haplotype scores (iHS) showing loci under positive selection. SNPs with |iHS| ≥ 3.14 (top 1%) are shown above the dashed horizontal line. Plot was generated by the R package *rehh*.

To investigate the potential evidence of drug selection, we evaluated the SNP diversity by measuring the average heterozygosity. We first assessed the effect of selective sweeps for the known resistance genes, *pfdhfr* and *pfcrt*. The parasites were grouped by their drug sensitivities, with resistant and sensitive groups being calculated for their levels of heterozygosity separately. We found clear selection signals in the region of *pfdhfr* and *pfcrt* where SNP diversity was reduced (Fig. 4a). Correspondingly, the extended haplotype homozygosity (EHH) analysis using alleles from 3D7 as the reference showed a slow decay of EHH in our samples around both the *pfdhfr* and *pfcrt* loci, further suggesting selective sweeps in those regions. Of the candidate resistant loci, we are particularly interested in the locus on chromosome 10, where the ATG18 and NIF4 genes are located and associated with decreased sensitivities to DHA, AM and the ACT-partner drug PPQ. When the parasites were grouped by their genotypes based on the significant SNP in ATG18, we indeed detected an extended region of ~22 kb around ATG18 where heterozygosity was reduced to 0, and the EHH decayed at a slower rate, suggesting there might be a recent selection (Fig. 4a3,b3). We then examined the genome-wide evidence for selection using integrated haplotype score (iHS). Using an iHS score of 3.14 as a significant cutoff value for the top 1% across the population, we detected multiple loci that were likely under significant positive selection (Fig. 5 and Supplementary Table S4). These included the malaria vaccine candidate genes *EBA-175*, *MSP1*, *AMA-1* and *TRAP*, consistent with previous observations in both Asian and African populations^34^,^42^,^43^,^44^. Positive selection also was detected at the PF3D7_0104100 locus, which was identified in previous selection scans in Southeast Asian and African populations^34^,^43^. This gene is located within 9.5 kb of *pfubp-1* (ubiquitin carboxyl-terminal hydrolase 1), the ortholog of which in the rodent parasite *P. chabaudi* was linked with ART resistance. Further, *pfubp-1* was associated with reduced susceptibility to ART in *P. falciparum* isolates from Kenya^43^. In addition, an autophagy protein ATG7 also was detected to be under positive selection. Signals for significant associations also were evident in *surfin 13.1* and *14.1*. However, direct selection on *pfcrt* and *pfdhfr* was not detected by iHS. This is because the iHS is defined as the standardized log-ratio of integrated EHH, which computes the integral of the observed decay of EHH away from a focal SNP until EHH reaches 0.05^45^. Although the EHH decay of those two regions from 1 to 0.1 was much slower in our isolates, it decayed faster from 0.1 to 0.05 than that in the reference strain (Fig. 4b1,b2), which resulted in the masking of potentially significant iHS scores.

## Discussion

GWAS has become an important approach for identifying molecular makers associated with genetic traits. In malaria parasites, genome-wide scan has been used to identify positive selections in the *P. falciparum* genome and markers for resistance to antimalarial drugs in different parasite populations^11^,^12^,^32^,^42^,^43^,^46^,^47^,^48^. In this study, we tested the association of SNPs from a high-density SNP array with *in vitro* susceptibilities to 10 antimalarial drugs in 94 *P. falciparum* parasites collected from the China-Myanmar border area, aiming to discover new genetic loci mediating and modifying drug sensitivities. This border region of China-Myanmar has a different antimalarial drug use history relative to other countries of the GMS such as Cambodia and Thailand. Besides, ART drugs have been used in this region for more than three decades mostly as monotherapies^27^, suggesting that different mutations mediating drug resistance might have been selected over time. The genetic background in these parasites is relatively uniform, and the confounding by a few outliers could be successfully corrected. To improve the confidence of the associations, we performed GWAS by use of multiple programs implemented with linear or nonparametric regression models. Furthermore, because of different distributions of the drug assay results, we used both original and transformed data for all GWAS analyses.

Extensive deployments of two antimalarials CQ and SP in the past in most malaria endemic areas have selected for high levels of resistance in parasite populations to these drugs. The principal genes *pfcrt* and *pfdhfr* associated with resistance to CQ and pyrimethamine, respectively, have been well characterized and are often used as the proof of principle in GWAS. In our *in vitro* drug assay, we found that the majority of parasites showed significant resistance to these drugs, and only several strains remained sensitive. The GWAS identified the most significant associations of CQ and SP resistance with *pfcrt* and *pfdhfr*, respectively. Although the genome-wide LD decays rapidly, strong LD was detected in a region of ~177 kb around *pfcrt*, where several genes were identified in the association analysis, consistent with a CQ selective sweep at the *pfcrt* locus. Encouraged by these results, we extended our analysis to eight additional antimalarial drugs. Five drugs had the same positive hits with at least two analysis programs, while PPQ had positive identifications by only one method.

Given the emergence and/or spread of ART resistance in many areas of the GMS, we performed GWAS using both the standard *in vitro* drug assay and the newly developed RSA. K13 mutations have been linked to clinical ART resistance in other regions of the GMS^3^,^13^,^41^. Using the RSA data, we identified significant association of a SNP in RAD5, a flanking gene of K13, with increased RSA values. In addition, GWAS also identified a region on chromosome 6, where the apicoplast translation protein EF-G and geranylgeranyltransferase are located. Both proteins have been indicated as potential drug targets and inhibition of their function has deleterious effects on parasites^49^,^50^. The identification of multiple loci associated with the RSA results may be correlated with the numerous potential cellular targets of the ART drugs^19^. Using data from the standard drug assay, two adjacent genes on chromosome 10, ATG18 and NIF4, were among the top list of genes significantly associated with IC_50_ data of both DHA and AM. NIF4 also was marginally associated with altered sensitivity to PPQ, a partner drug of ACT which has been used extensively in China as a replacement drug of CQ. Furthermore, reduced heterozygosity and delayed EHH at this locus suggested a recent selection in this region. Interestingly, NIF4 also showed significant association with DPC in parasites from other location of Southeast Asia. Noteworthy, an ART-based probe covalently bound to ATG18, suggesting it might be an ART target^19^. Further, since ATG18 binding to PI3P is required for robust autophagic activity in yeast^51^, regulated autophagy might play a role in parasite’s response to oxidative stress imposed by certain antimalarial drugs^52^. Recently, strong evidence suggests that the PI3K signaling pathway is a target of ART, and several K13 mutations were linked to increased activity of PI3K and the corresponding PI3P level^17^. Thus, the association of ATG18 mutation with reduced sensitivities to AM and DHA in the standard IC_50_ assay presents a potential, logical link between the autophagy and PI3K pathways in mediating ART resistance.

To detect genome-wide loci potentially under recent positive selection we employed the iHS as a measure of long-range directional selection. Consistent with earlier studies^42^,^43^, this analysis identified several genes involved in merozoite invasion, which are among the top vaccine candidates. It is well known that these genes are under strong balancing selection by host immunity. Of particular interest is the detection of PF3D7_0104100, a gene adjacent to the deubiquitinating protease gene *pfubp1*. Association of *ubp1* with ART resistance was first identified in *P. chabaudi* and later identified in *P. falciparum*^43^,^53^. The involvement of ubiquitination-proteasome pathway in ART resistance may imply a potential role of *ubp1* in stress response^18^. In Kenya, though the mutant *pfubp1* was not associated with an increased risk of subsequent recurrence of infection post ACT treatment, the mutant genotype was found to be significantly more prevalent in recurrent samples^54^. In Cambodia where clinical ART resistance has been detected, selection on the *pfubp1* gene was not detected^41^, suggesting that the impact of *ubp1* may be dependent on parasite genetic backgrounds. The positive selection around *pfcrt* and *dhfr*, which was detected by EHH, was not identified by iHS due to the faster decay of EHH from 0.1 to 0.05 in field isolates than that in the reference strain. In addition, we also did not detect long-range selection in other drug resistance gene loci such as K13 and *pfmdr1* in our parasite population. This could be due to that the selection on K13 by the ART drugs might be a recent event^55^. As a marker for multidrug resistance, *pfmdr1* mutations have been linked to resistance to MQ, AS, and QN in field parasites and confirmed by genetic manipulations^56^,^57^. Extensive use of MQ in Thailand and Cambodia has selected for increased copy numbers of *pfmdr1*^58^. As such, selective sweep has been observed in parasite populations from this region^59^. It is likely that the lack of a selective sweep in the *pfmdr1* locus in the parasite population from the China-Myanmar border area is due to no previous MQ usage in this region. Likewise, our earlier studies only detected Y184F mutation but no *pfmdr1* amplification in parasites from this region^10^,^29^.

In this study we used robust *in vitro* drug assay data from 94 culture-adapted parasites from the GMS as phenotypes in GWAS and identified new candidate genes associated with *in vitro* drug resistance. Though the parasites exhibited >10 fold difference in their sensitivities to the 10 drugs tested, we did not identify a considerable number of SNPs associated with *in vitro* resistance for certain drugs (e.g., MQ, AS, PPQ and LMF). In addition, the rapid decay of LD within several hundred base pairs in *P. falciparum* somewhat limits the power of GWAS with the SNP array data. An increase of population size and use of genome sequencing data are likely to increase the power of the GWAS. This also would allow us to detect the linkage of resistant parasites to genetic background, which may predispose parasites to the evolution of resistance^41^.

## Materials and Methods

### Parasite collection and culture adaption

*Plasmodium falciparum* infected blood samples were collected during the years 2004–2011 from febrile patients attending local clinics located along the China-Myanmar border. Uncomplicated *P. falciparum* malaria was identified by microscopy. The study has been approved by the Institutional Review Boards of Penn State University, Kunming Medical University and Department of Health of Kachin, and informed consent was obtained from all adult participants and from parent/guardian of children. The collecting and processing of venous blood samples were performed in accordance with the Appropriate Technology in Health (PATH) guidelines. A total of 94 parasite isolates (2 in 2004, 8 in 2007; 15 in 2008; 58 in 2009; 8 in 2010 and 4 in 2011), determined to be monoclonal infections by genotyping three polymorphic antigen markers *msp1*, *msp2* and *glutamate-rich protein*, were retrieved from an archive of culture-adapted parasites and used for *in vitro* drug assay and genotyping. Routine cultures of the parasites were maintained in type O^+^ human red blood cells in complete medium supplemented with 6% human AB serum under an atmosphere of 90% N_2_/5% O_2_/5% CO_2_.

### *In vitro* drug assay and IC_50_ calculation

The standard SYBR Green I-based fluorescence assay was used to assess parasite susceptibilities to 10 antimalarial drugs: DHA, AS, AM, PND, LMF, PPQ, MQ, CQ, QN and SP (S:P = 20:1, w/w), of which CQ and SP served as internal controls to validate the analysis. DHA, AS, AM, MQ, CQ, QN, and SP were purchased from Sigma (St. Louis, MO, USA), while PND, LMF and PPQ were obtained from Kunming Pharmaceutical Co. (Kunming, Yunnan, China). The stock solution of AM was dissolved in dimethyl sulfoxide (DMSO), and SP in 40% DMSO. Others were prepared as previous described^10^. *In vitro* cultures were synchronized with two rounds of 5% D-sorbitol treatment, and ring-stage parasites were assayed for drug sensitivity in 96-well microtiter plates at 1% hematocrit and 0.5% parasitemia. Three biological replicates were tested for each isolate and each drug concentration was repeated twice. To reduce the variations between plates, the standard laboratory clone 3D7 was always included as a reference. RSA was performed to measure susceptibility of ring-stage parasites to DHA following the INV10 Standard Operating Procedure^10^. IC_50_ was measured using a non-linear regression model in GraphPad Prism 5 (GraphPad Software, Inc. La Jolla, CA, USA). Normal distribution of the assay values was tested by Shapiro-Wilk test. Geometric mean of the IC_50_ and 95% confidence interval were calculated for normally distributed data. Median and interquartile range were calculated if the data were not normally distributed. The correlation between drug assays was assessed by Spearman’s correlation coefficients in R. Student *t*-test was applied to investigate potentially significant differences in mean assay values between the field isolates and 3D7.

### SNP array and data imputation

Parasite genomic DNA was extracted from cultured isolates using Wizard® Genomic DNA Purification Kit (Promega, WI, USA). Genomic DNA was genotyped utilizing a high-density Affymetrix SNP array that allows interrogation of over 17,000 SNPs performed at RML Genomics Unit, Research Technologies Branch, NIAID. The BRLMM-P algorithm was used to call the SNPs as previously reported^32^. Genotypes were transferred to a spreadsheet for further analyses. SNPs within *var*, *rifin* and *stevor* genes were excluded to reduce artifacts due to duplicated sequences. SNPs with call rates below 90% were also removed. The SNP data were then imputed by using the software BEAGLE v3.3.2^33^. This dataset was further filtered to remove multi-allelic and low-frequency SNPs with MAF of <2% by PLINK 1.9.

### LD measurement and population structure determination

The genome-wide pairwise LD was measured by PLINK 1.9, setting a flag of -ld-window-r^2^ to 0 to include all pairs of SNPs. A setting of this flag to 0.3 was used to measure the SNP pairs with R^2^ > 0.3. LD values were plotted by using the R software suite. Population structure was investigated by PCA based on the variance-standardized relationship matrix using PLINK 1.9.

### GWAS

Phenotypes were transformed by natural logarithm and rank-based inverse-normal transformation. To account for zeros in the RSA results, 0.5% was added to all values for log-transformation. GWAS was performed using multiple software packages, GEMMA, PLINK 1.9, linear-regression and nonparametric regression in R. In addition, the newly developed WarpedLMM that estimates optimal transformation of the phenotypes was also applied to the analysis. For PLINK, R and WarpedLMM, the top three principal components as covariates were applied to correct the inflation and reduce the influences of population structure. GEMMA estimates genetic relatedness from genotypes and automatically adjusts the inflation. *P* values were obtained from each model and a threshold after Bonferroni correction (0.05/number of SNPs analyzed) was used to assess the genome-wide significance. Q-Q plots for *P* values were used to evaluate the robustness of different models in minimizing inflation due to population stratification. The genomic inflation factor lambda (λ) was calculated for each drug susceptibility phenotype by an R package *snpStats*. Manhattan plots were generated by modified scripts from the R package *CMplot*.

### SNP diversity analysis

Genetic diversity at resistance loci was evaluated by the average heterozygosity. We calculated the expected heterozygosity using the equation 
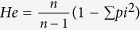
, where *He* is the expected heterozygosity, *n* is the sample size and *pi* is the frequency of the *i*th allele. The heterozygosity values were averaged by gene, and if there was only one SNP in a gene or no annotation for one SNP, it was grouped in the neighbor gene within 10 kb.

### Recent directional selection

Positive selection at resistance loci was examined by EHH in the R package *rehh*^60^, a method that detects the transmission of an extended haplotype without recombination. The SNPs at S220A in *pfcrt*, C59R in *pfdhfr* and T38N in *pfatg18* served as the focal SNPs in the EHH analysis. The iHS, which was also implemented in *rehh*, was used to identify genome-wide long-range directional selection. The iHS is the standardized log-ratio of the integrated extended-haplotype homozygosity for the ancestral and derived alleles at a core SNP, with large positive values indicating long haplotypes carrying the ancestral allele and large negative values indicating long haplotypes carrying the derived allele. Both extreme positive and negative iHS scores are potentially interesting, since ancestral alleles may hitchhike with selected site having large positive score^45^. Therefore, we used the absolute values of iHS to capture unusually long haplotypes surrounding both types of alleles.

## Additional Information

**How to cite this article**: Wang, Z. *et al.* Genome-wide association analysis identifies genetic loci associated with resistance to multiple antimalarials in *Plasmodium falciparum* from China-Myanmar border. *Sci. Rep.* **6**, 33891; doi: 10.1038/srep33891 (2016).

## Supplementary Material

Supplementary Information

## Figures and Tables

**Table 1:** Significant associations with six antimalarial drugs.

	Gene ID	Chr.	Position	Function	No. SNPs
DHA	null	7	152148	RWD domain-containing protein	1
	PF3D7_0823100	8	1019263		1
	PF3D7_1012700	10	490648	NLI interacting factor-like phosphatase (NIF4)	2
	PF3D7_1012900	10	497461	autophagy-related protein 18 (ATG18)	1
AM	null	4	976619	conserved Plasmodium protein	1
	PF3D7_0805300	8	294826		1
	PF3D7_1012700	10	490648	NLI interacting factor-like phosphatase (NIF4)	2
	PF3D7_1012900	10	497461	autophagy-related protein 18, (ATG18)	1
PND	PF3D7_1014100	10	557657	conserved Plasmodium protein	1
	PF3D7_1023700	10	992490	conserved Plasmodium protein	1
	PF3D7_1138000	11	1493767	conserved Plasmodium protein	1
	PF3D7_1201900	12	107560	conserved protein	1
	PF3D7_1308900	13	409854	mRNA-decapping enzyme 2 (DCP2)	1
LMF	PF3D7_1148800	11	1941896	Plasmodium exported protein (hyp11)	1
QN	PF3D7_0205800	2	233242	conserved Plasmodium protein	1
	null	8	8211		1
	null	12	1776215		1
	PF3D7_1305300	13	263239	conserved Plasmodium protein	1
	PF3D7_1309200	13	426280	protein phosphatase PPM6, putative (PPM6)	1
	PF3D7_1313100	13	564975	conserved Plasmodium protein	1
	PF3D7_1467600	14	2757814	conserved Plasmodium protein	1
PPQ	PF3D7_0804900	8	280180	GTPase-activating protein	1
	null	11	77735		1
	null	12	1959796		1
DHA^RSA^	PF3D7_0102500	1	114712	erythrocyte binding antigen-181 (EBA181)	1
	null	2	60099		1
	null	2	62496		1
	PF3D7_0205800	2	233242	conserved Plasmodium protein	1
	PF3D7_0216600	2	687214	MtN3-like protein	1
	PF3D7_0220800	2	840798	cytoadherence linked asexual protein 2 (CLAG2)	1
	PF3D7_0319700	3	829388	ABC transporter I family member 1 (ABCI3)	1
	PF3D7_0323700	3	992785	U4/U6.U5 tri-snRNP-associated protein 1 (SART1)	1
	null	4	163735		1
	PF3D7_0410800	4	489105	conserved Plasmodium protein	1
	null	4	496634		1
	PF3D7_0501800	5	92998	chromosome assembly factor 1 (CAF1)	1
	PF3D7_0522400	5	923033	conserved Plasmodium protein	1
	PF3D7_0522900	5	952155	zinc finger protein	1
	PF3D7_0602400	6	103660	elongation factor G (EF-G)	1
	PF3D7_0602500	6	105830	geranylgeranyltransferase	1
	PF3D7_0605600	6	232370	nucleoside diphosphate kinase	1
	null	6	1300041		1
	PF3D7_0726200	7	1100698	serine/threonine protein kinase, FIKK family (FIKK7.1)	1
	PF3D7_0728100	7	1203807	conserved Plasmodium membrane protein	1
	PF3D7_0808000	8	405667	conserved Plasmodium protein	1
	PF3D7_0823600	8	1041193	lipoate-protein ligase B (LipB)	1
	PF3D7_0827100	8	1174019	translation initiation factor IF-2 (IF2c)	1
	null	9	428865		1
	null	9	510573		1
	null	9	1155698		1
	null	10	260411		1
	PF3D7_1018100	10	721205	conserved Plasmodium protein	1
	PF3D7_1029600	10	1206581	adenosine deaminase (ADA)	1
	PF3D7_1106500	11	274072	conserved Plasmodium protein	1
	PF3D7_1131000	11	1194588	RNA-binding protein s1, putative	1
	PF3D7_1131400	11	1206440	conserved Plasmodium protein	1
	PF3D7_1205100	12	223538	O-phosphoseryl-tRNA (Sec) selenium transferase (SEPSECS)	1
	null	12	2218016		1
	PF3D7_1309200	13	426280	protein phosphatase PPM6 (PPM6)	1
	PF3D7_1328200	13	1191385	conserved Plasmodium protein	1
	PF3D7_1329100	13	1229678	myosin C (MyoC)	2
	PF3D7_1343400	13	1718288	DNA repair protein RAD5 (RAD5)	1
	PF3D7_1411000.2	14	443123	conserved Plasmodium protein	1
	PF3D7_1423700	14	959160	conserved Plasmodium protein	1
	PF3D7_1439500	14	1609726	CCAAT-binding transcription factor	1
	PF3D7_1453500	14	2196699	pyridine nucleotide transhydrogenase	1
	null	14	2410693		1
	PF3D7_1467200	14	2748665	WD repeat-containing protein 79	1

See Table S4 for associations with CQ and SP, and detailed *P*-values. Chr., chromosome; dihydroartemisinin (DHA); artemether (AM); piperaquine (PPQ); quinine (QN), lumefantrine (LMF); and pyronaridine (PND); ring-survival rates from RSA (DHA^RSA^).
