# Supplementary material for: Genome-wide association analysis identifies genetic loci associated with resistance to multiple antimalarials in Plasmodium falciparum from China-Myanmar border
Source: Sci Rep. 2016 Oct 3;6:33891. doi: 10.1038/srep33891 (PMC5046179; doi:10.1038/srep33891)
Supplement: Supplementary Information [file srep33891-s1.pdf]

Supplementary Materials for

Genome-wide association analysis identifies genetic loci associated with resistance to multiple antimalarials in *Plasmodium falciparum* from China-Myanmar border

Zenglei Wang, Mynthia Cabrera, Jingyun Yang, Lili Yuan, Bhavna Gupta, Xiaoying Liang, Karen Kemirembe, Sony Shrestha, Awtum Brashear, Xiaolian Li, Stephen F. Porcella, Jun Miao, Zhaoqing Yang, Xin-zhuan Su & Liwang Cui*

*Correspondence to: Liwang Cui (luc2@psu.edu)

This PDF file includes:

Figure S1: Distribution of SNPs according to minor allele frequency (MAF).

Figure S2: Distributions of SNPs after quality control and the average physical distance between adjacent SNPs on each chromosome.

Figure S3: Strong linkage disequilibrium with $R^2 > 0.3$ in the entire population.

Figure S4: Quantile-quantile (Q-Q) plots after PCA correction.

Figure S5: Manhattan plots of the genome-wide association tests.

Table S1: *In vitro* IC50s (nM/μg/ml*) to 10 antimalarial drugs and ring-stage survival rates of 3D7 and 94 culture-adapted clinical isolates.

Table S2: Genomic loci significantly associated with altered drug sensitivities identified by GWAS.

Table S3: Pairwise LD of SNPs at the *pfcrt*, *dhfr*, *atg18* and *nif4* genome loci ($R^2 > 0.3$).

Table S4: SNPs/genes under significant positive selection detected using integrated haplotype scores (iHS).

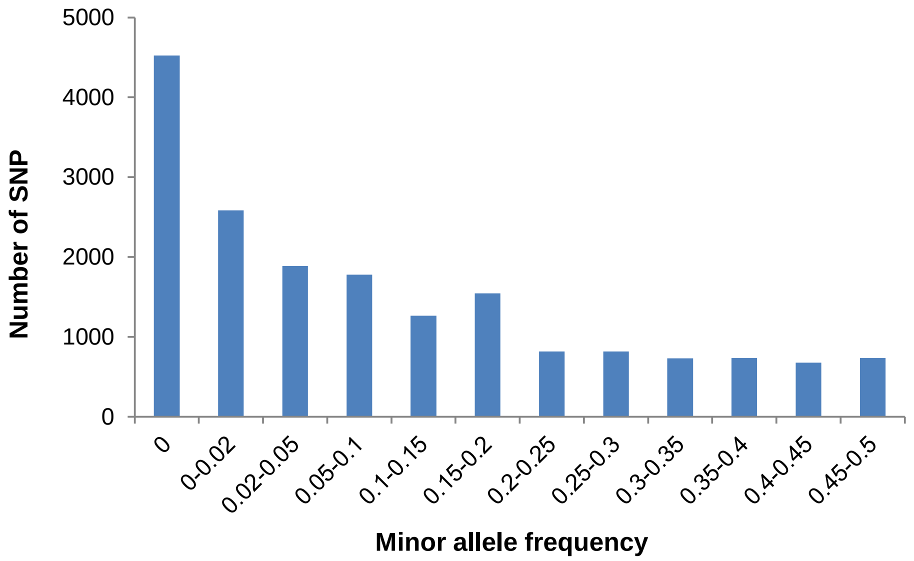

Figure S1. Distribution of SNPs according to minor allele frequency (MAF). There is an overabundance of low-frequency SNPs (MAF<5%).

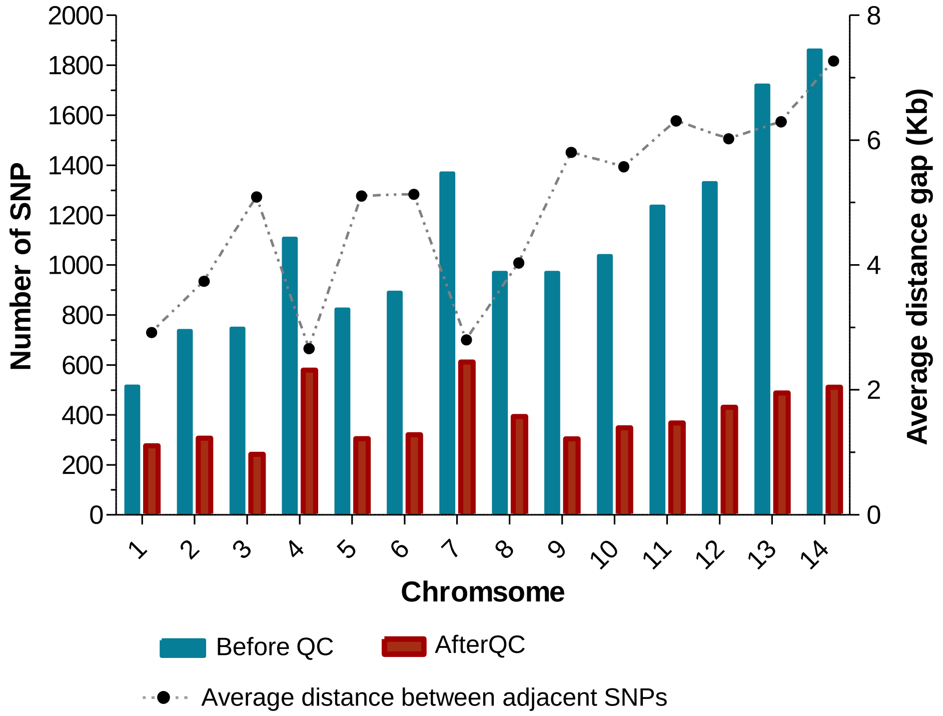

Figure S2. Distributions of SNPs after quality control and the average physical distance between adjacent SNPs on each chromosome.

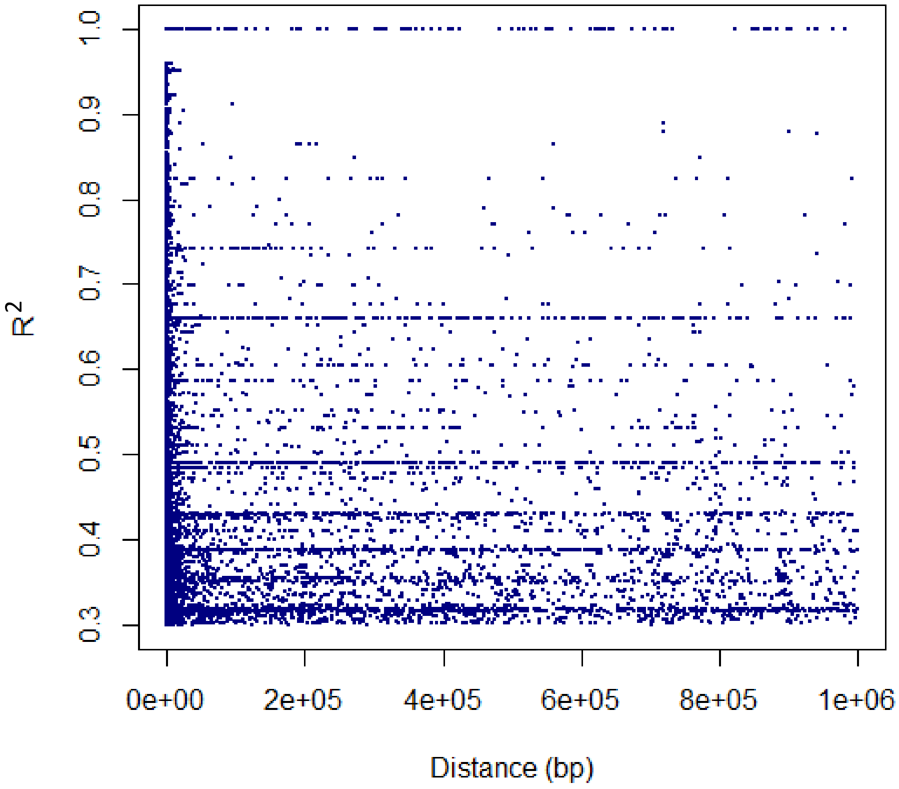

Figure S3. Strong linkage disequilibrium determined as squared correlation of allele frequencies (R^2) larger than 0.3 against physical map distance (bp) between linked loci pairs in the entire population.

A.

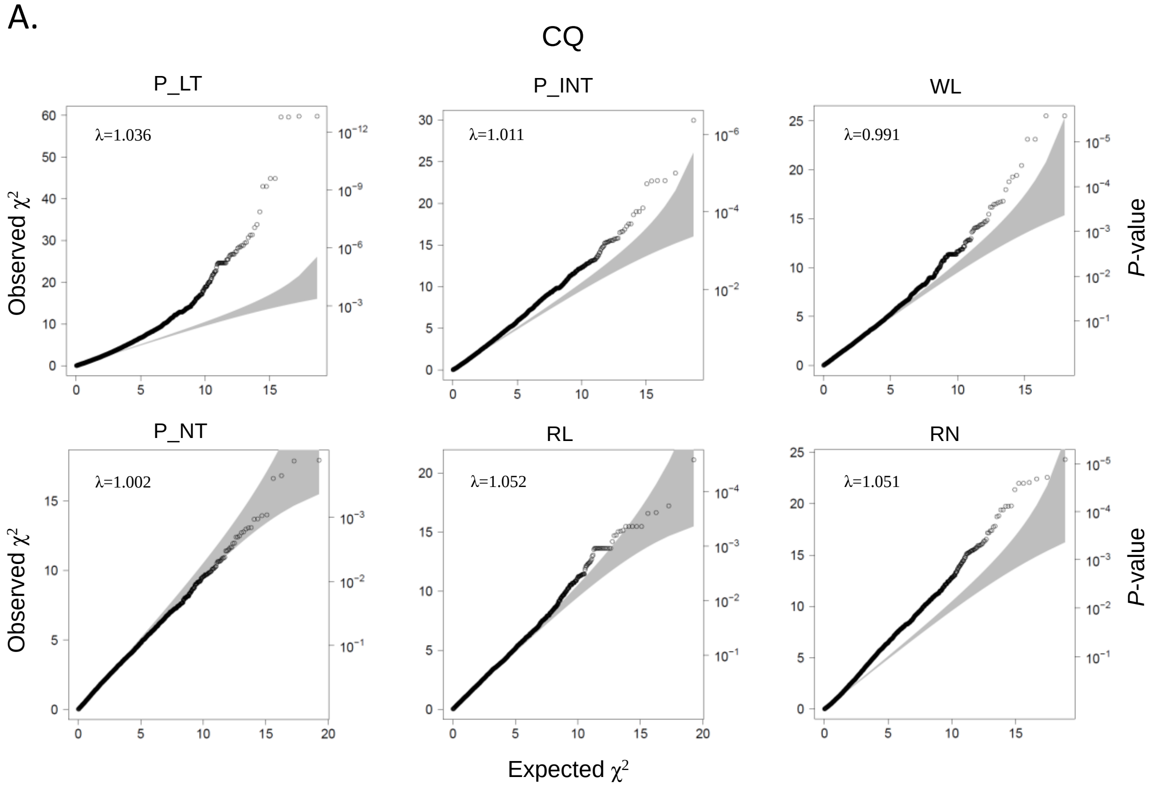
CQ
P_LT
λ=1.036
P_INT
λ=1.011
WL
λ=0.991
P_NT
λ=1.002
RL
λ=1.052
RN
λ=1.051
Observed χ2
P-value
Expected χ2

B.

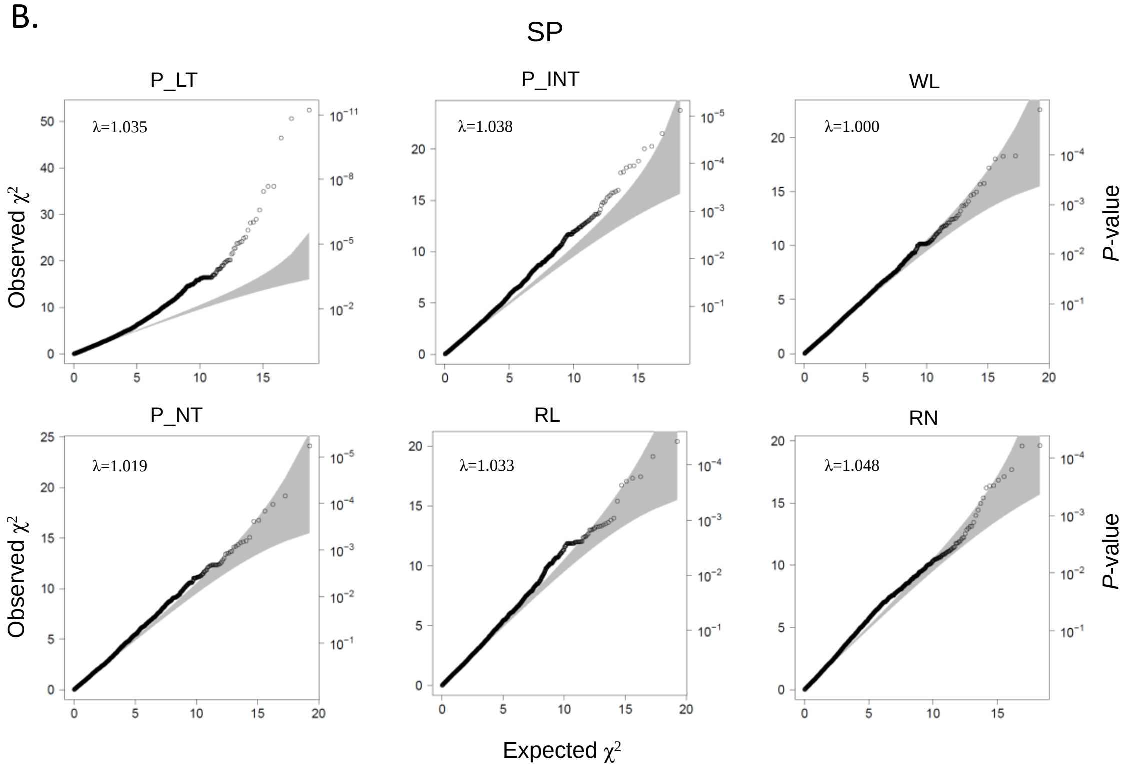
SP
P_LT
λ=1.035
P_INT
λ=1.038
WL
λ=1.000
P_NT
λ=1.019
RL
λ=1.033
RN
λ=1.048
Observed χ2
P-value
Expected χ2

C.

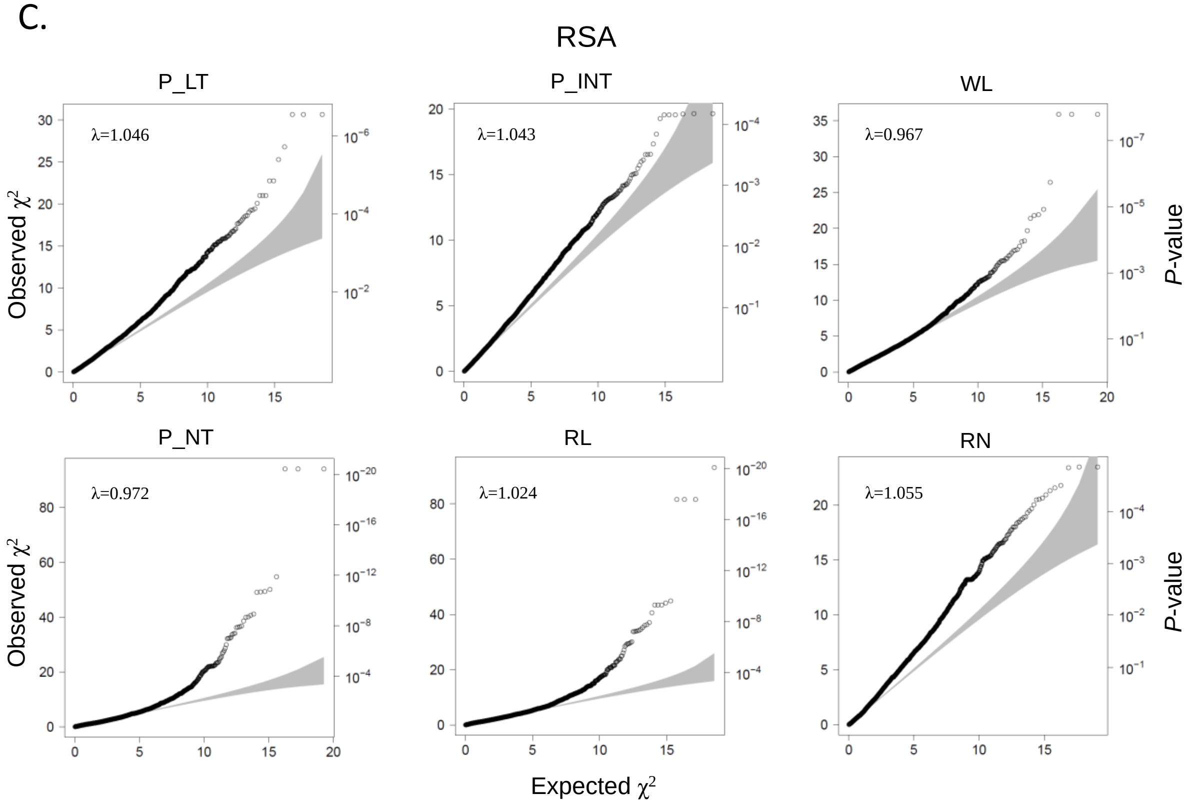
RSA
P_LT
λ=1.046
P_INT
λ=1.043
WL
λ=0.967
P_NT
λ=0.972
RL
λ=1.024
RN
λ=1.055
Observed χ2
P-value
Expected χ2

D.

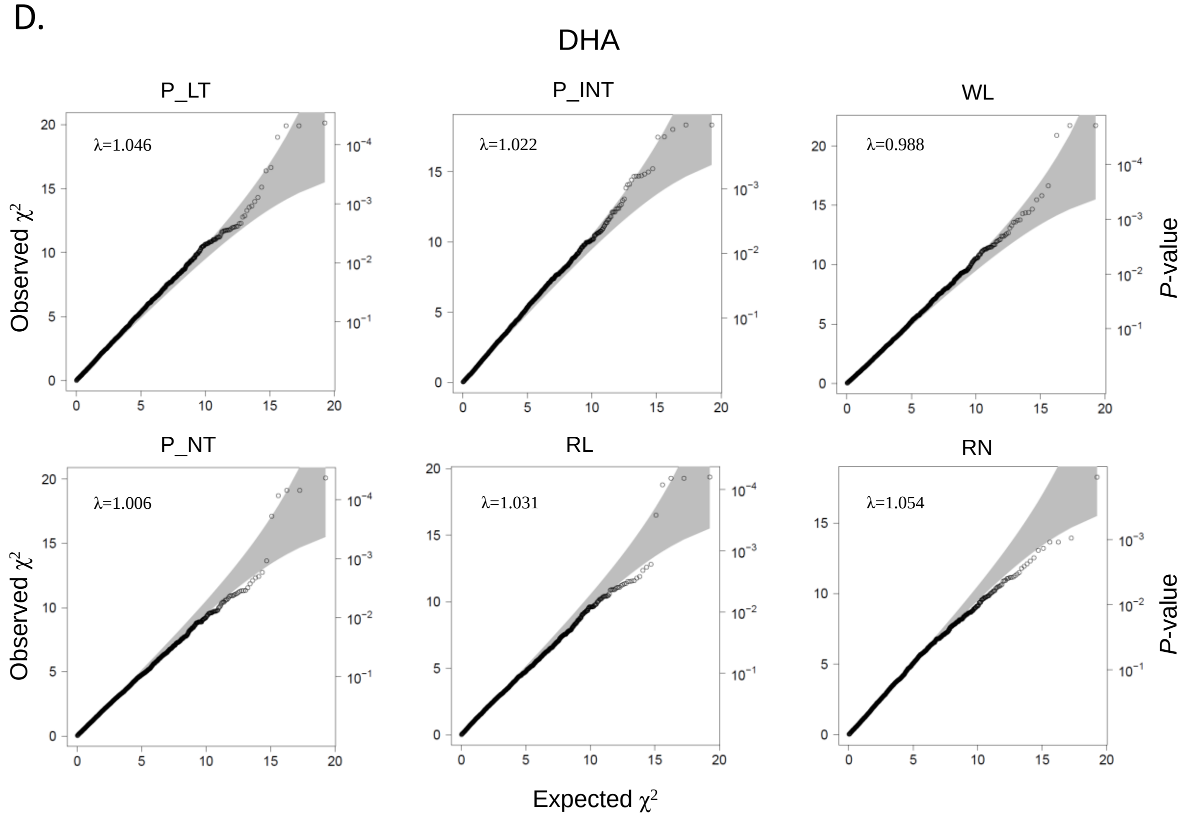
DHA
P_LT
λ=1.046
P_INT
λ=1.022
WL
λ=0.988
P_NT
λ=1.006
RL
λ=1.031
RN
λ=1.054
Observed χ2
P-value
Expected χ2

E.

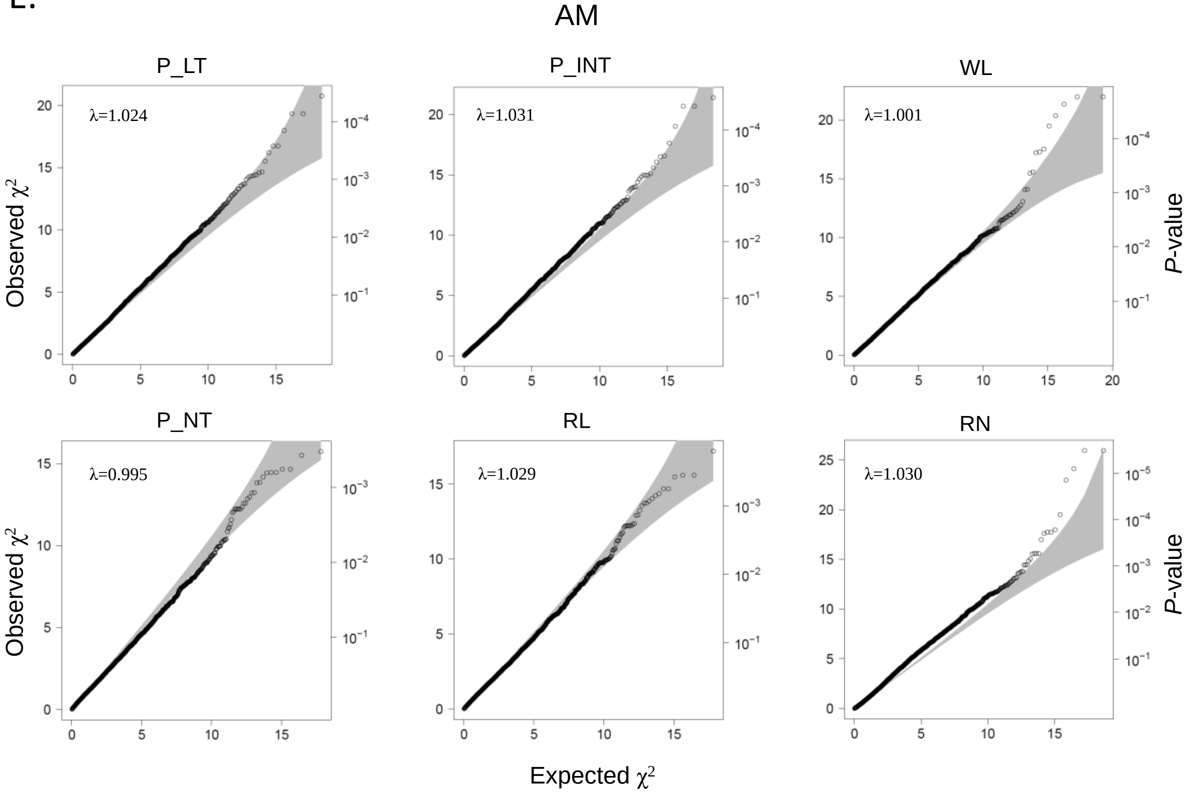
AM
P_LT
λ=1.024
P_INT
λ=1.031
WL
λ=1.001
P_NT
λ=0.995
RL
λ=1.029
RN
λ=1.030
Observed χ2
Expected χ2
P-value

F.

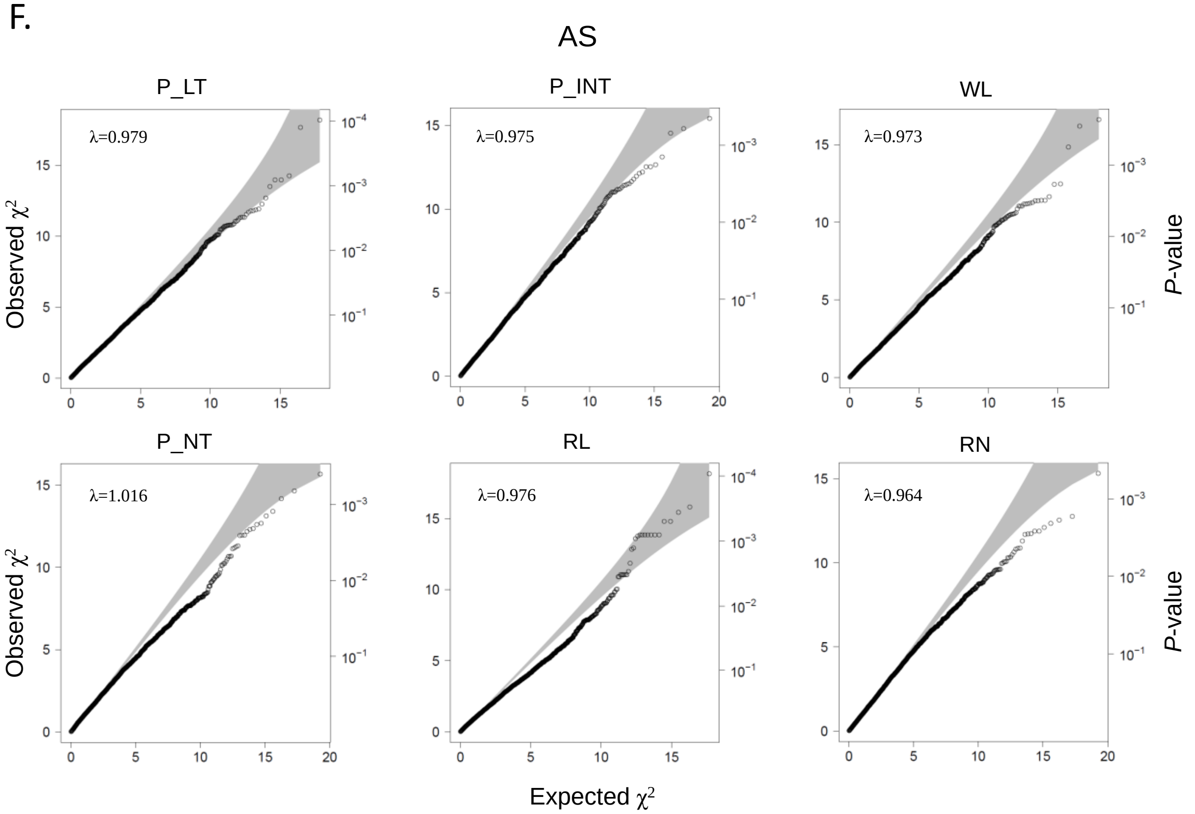
AS
P_LT
λ=0.979
P_INT
λ=0.975
WL
λ=0.973
P_NT
λ=1.016
RL
λ=0.976
RN
λ=0.964
Observed χ2
Expected χ2
P-value

G.

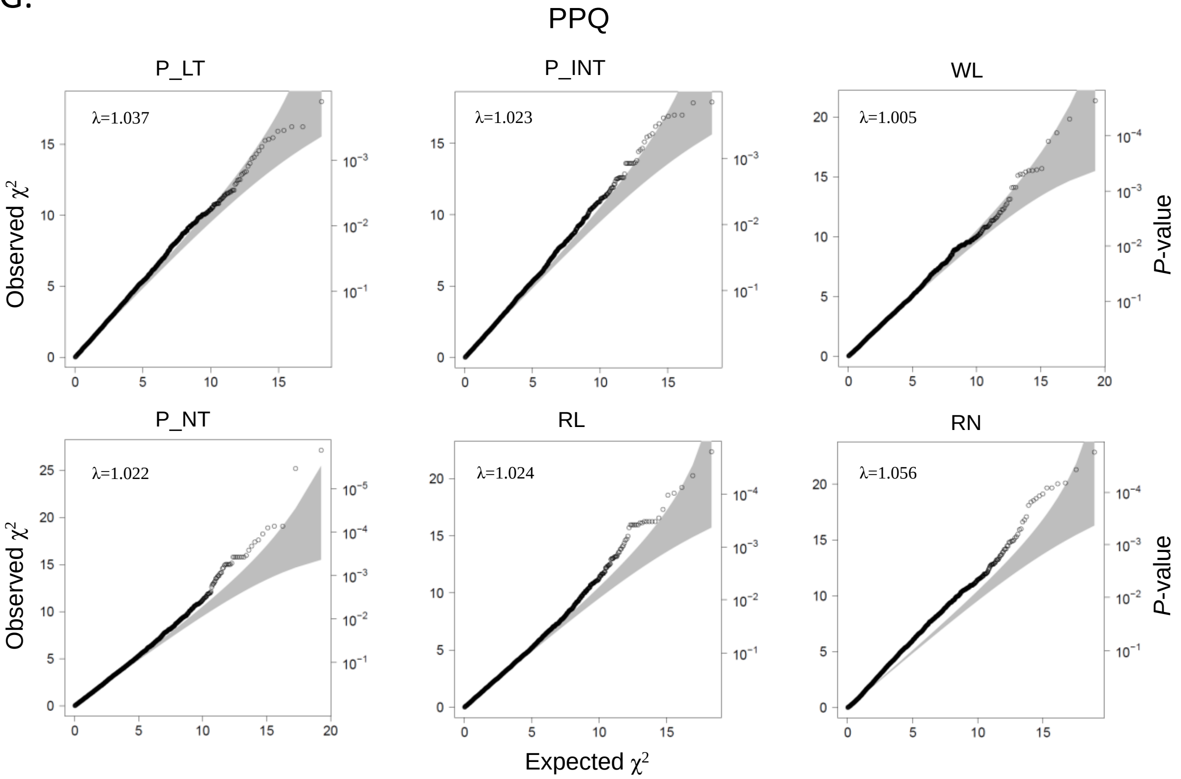
PPQ
P_LT
λ=1.037
P_INT
λ=1.023
WL
λ=1.005
P_NT
λ=1.022
RL
λ=1.024
RN
λ=1.056
Observed χ2
P-value
Expected χ2

H.

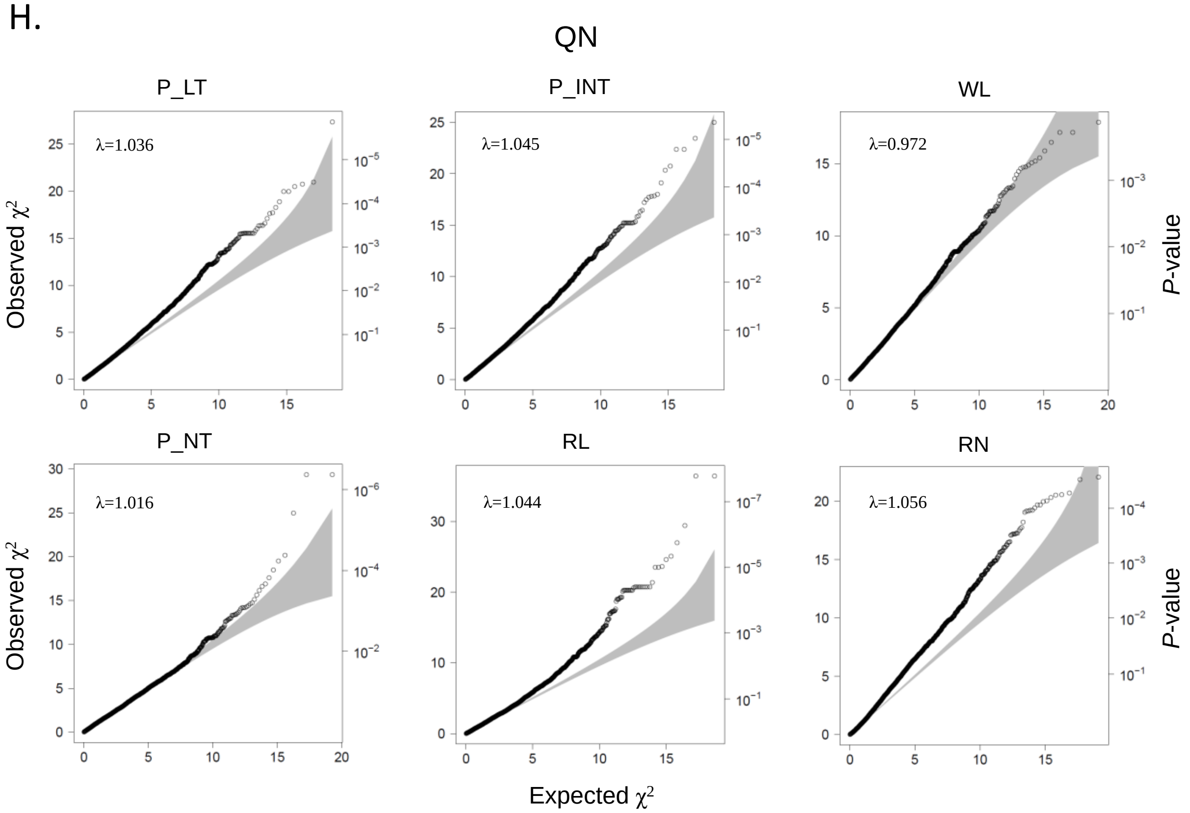
QN
P_LT
λ=1.036
P_INT
λ=1.045
WL
λ=0.972
P_NT
λ=1.016
RL
λ=1.044
RN
λ=1.056
Observed χ2
P-value
Expected χ2

I.

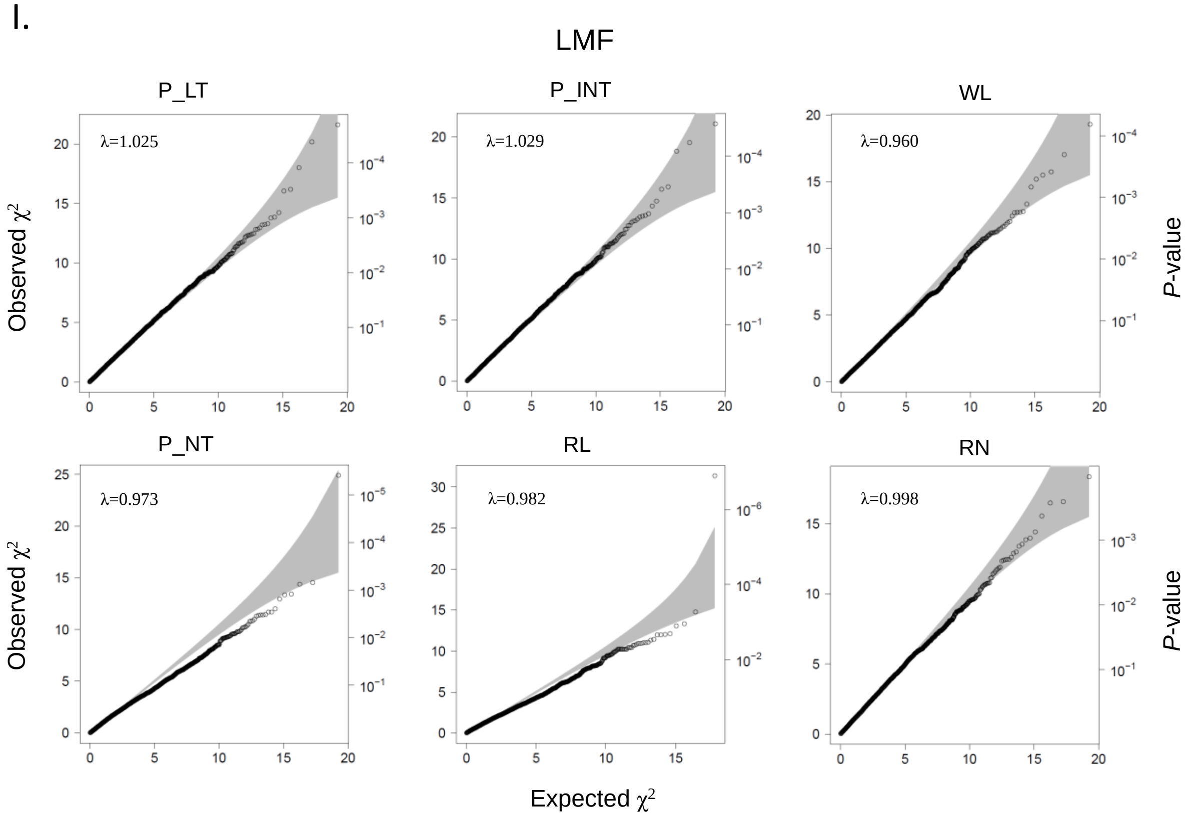
LMF
P_LT
λ=1.025
P_INT
λ=1.029
WL
λ=0.960
P_NT
λ=0.973
RL
λ=0.982
RN
λ=0.998
Observed χ²
P-value
Expected χ²

J.

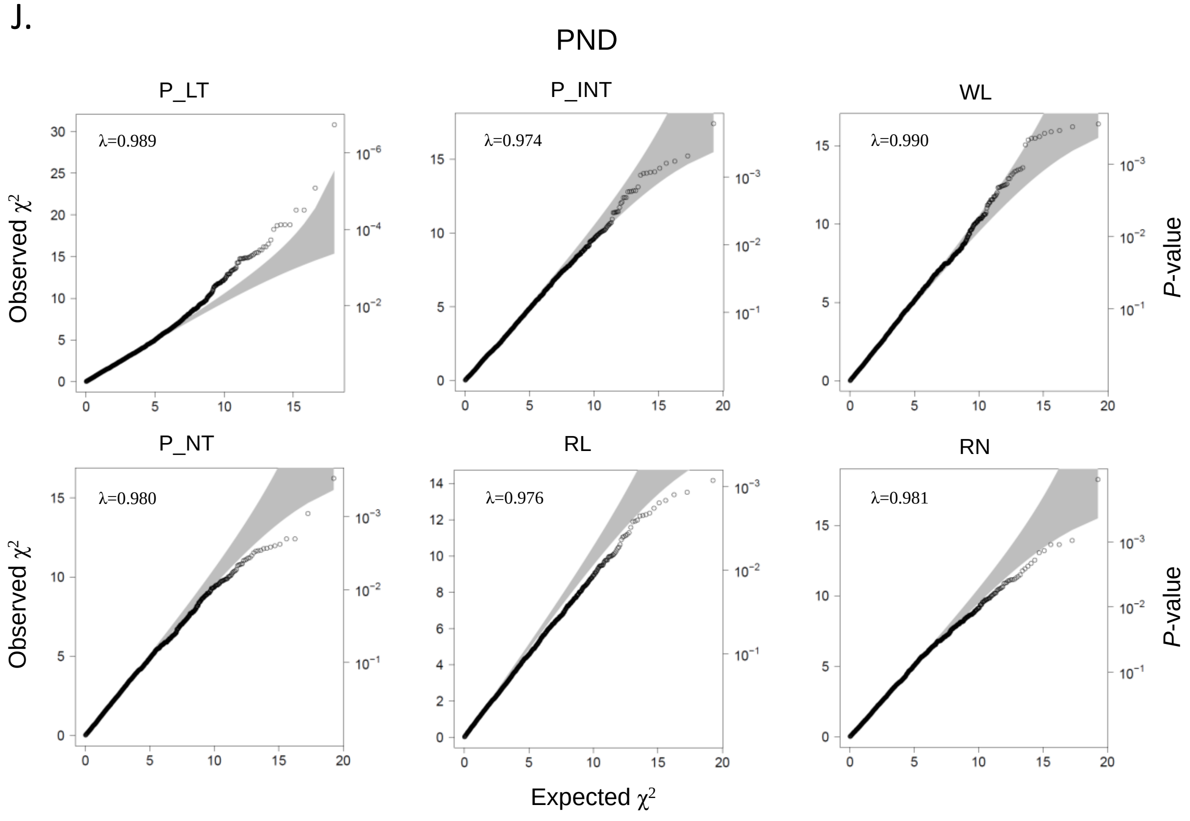
PND
P_LT
λ=0.989
P_INT
λ=0.974
WL
λ=0.990
P_NT
λ=0.980
RL
λ=0.976
RN
λ=0.981
Observed χ²
P-value
Expected χ²

K.

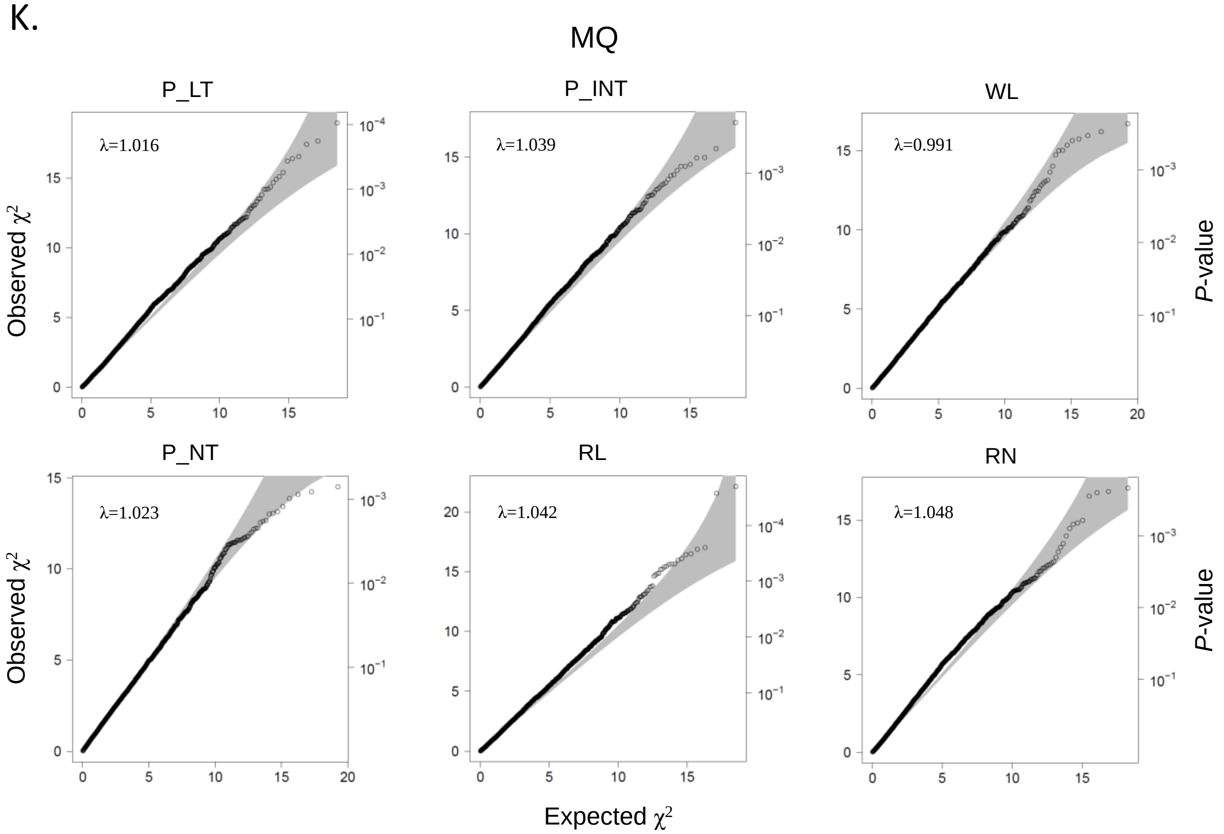

Figure S4. Quantile-quantile (Q-Q) plots after PCA correction. Q-Q plots were generated by plotting the expected chi-squared values (X-axis) against the observed chi-squared values (left Y-axis) by the R package *snpStats* to estimate the extent of association test statistic inflation. The *P*-values were shown in right Y-axis. Black dots represent the observed χ^2, and the grey area is the expected 95% confident intervals under the null distribution. The genomic inflation factor, lambda (λ), was used to indicate structure inflation. Data from 10 drugs are presented, with *A* for chloroquine (CQ), *B* for sulfadoxine-pyrimethamine (SP), *C* for ring-stage survival rates from the ring-stage survival assay, *D* for dihydroartemisinin (DHA), *E* for artemether (AM), *F* for artesunate (AS), *G* for piperaquine (PPQ), *H* for quinine (QN), *I* for lumefantrine (LMF), *J* for pyronaridine (PND) and *K* for mefloquine (MQ). Q-Q plots of results from Plink and R programs are shown. P_LT: Plink with log-transformed phenotypes; P_INT: Plink with INT-transformed phenotypes; WL: WarpedLMM; P_NT: Plink with non-transformed phenotypes; RL: linear regression in R with non-transformed phenotypes; RN: non-parametric regression in R with non-transformed phenotypes. Inflation in GEMMA was automatically adjusted and thus the Q-Q plots are not shown.

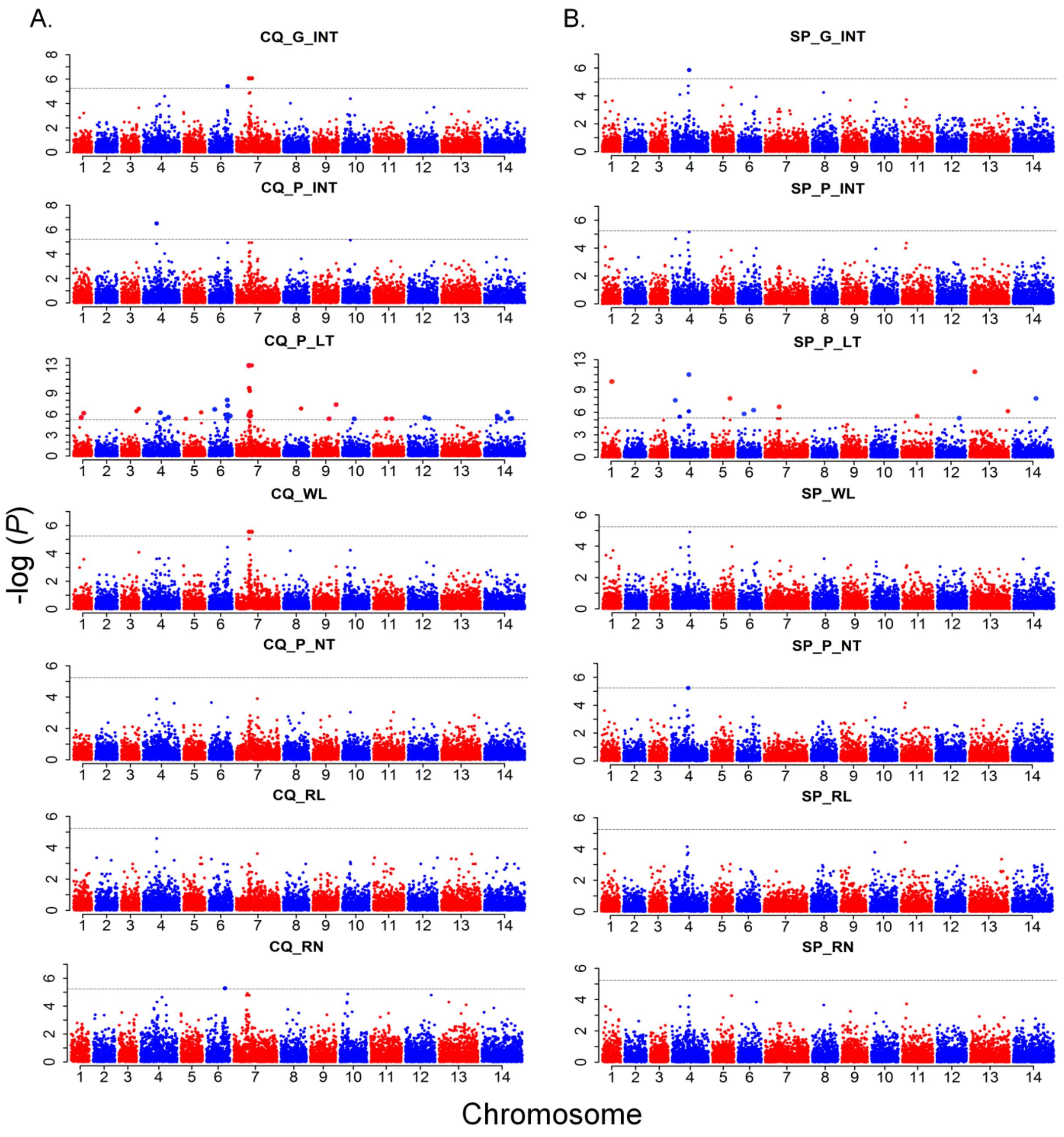
A.
B.
CQ_G_INT
CQ_P_INT
CQ_P_LT
CQ_WL
CQ_P_NT
CQ_RL
CQ_RN
SP_G_INT
SP_P_INT
SP_P_LT
SP_WL
SP_P_NT
SP_RL
SP_RN
-log (P)
Chromosome

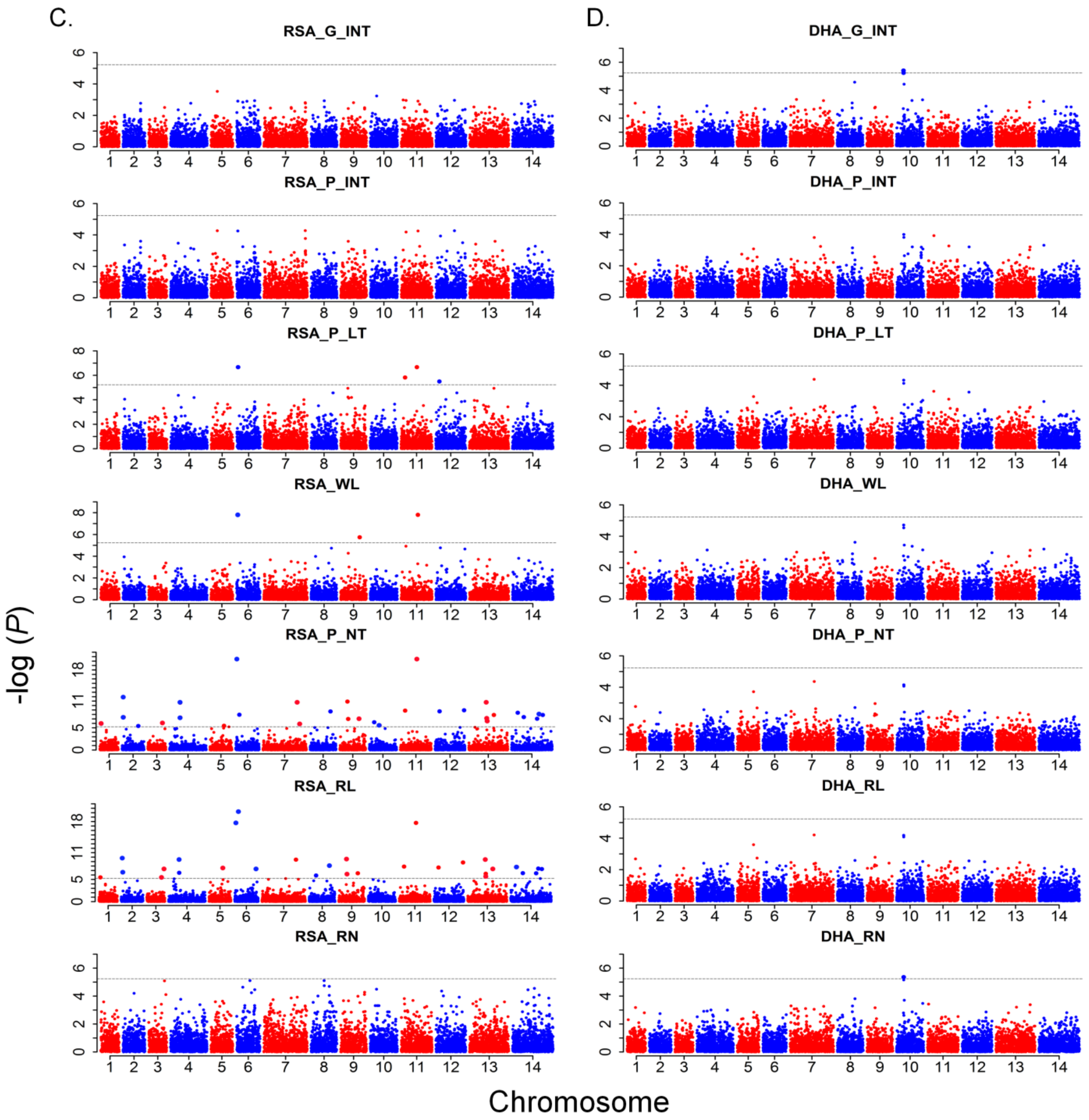
C.
D.
RSA_G_INT
RSA_P_INT
RSA_P_LT
RSA_WL
RSA_P_NT
RSA_RL
RSA_RN
DHA_G_INT
DHA_P_INT
DHA_P_LT
DHA_WL
DHA_P_NT
DHA_RL
DHA_RN
-log (P)
Chromosome

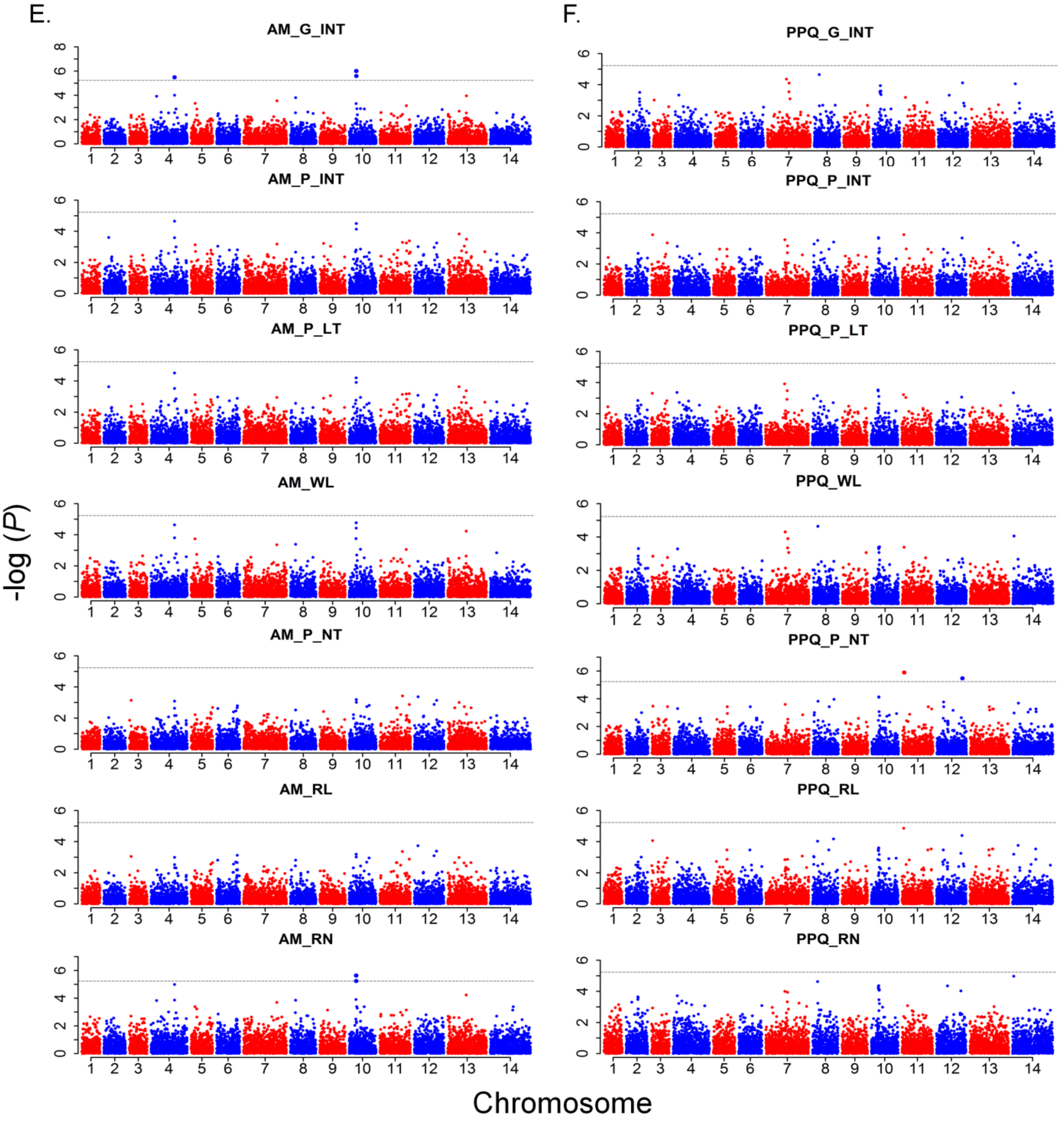
E.
F.
AM_G_INT
AM_P_INT
AM_P_LT
AM_WL
AM_P_NT
AM_RL
AM_RN
PPQ_G_INT
PPQ_P_INT
PPQ_P_LT
PPQ_WL
PPQ_P_NT
PPQ_RL
PPQ_RN
-log (P)
Chromosome

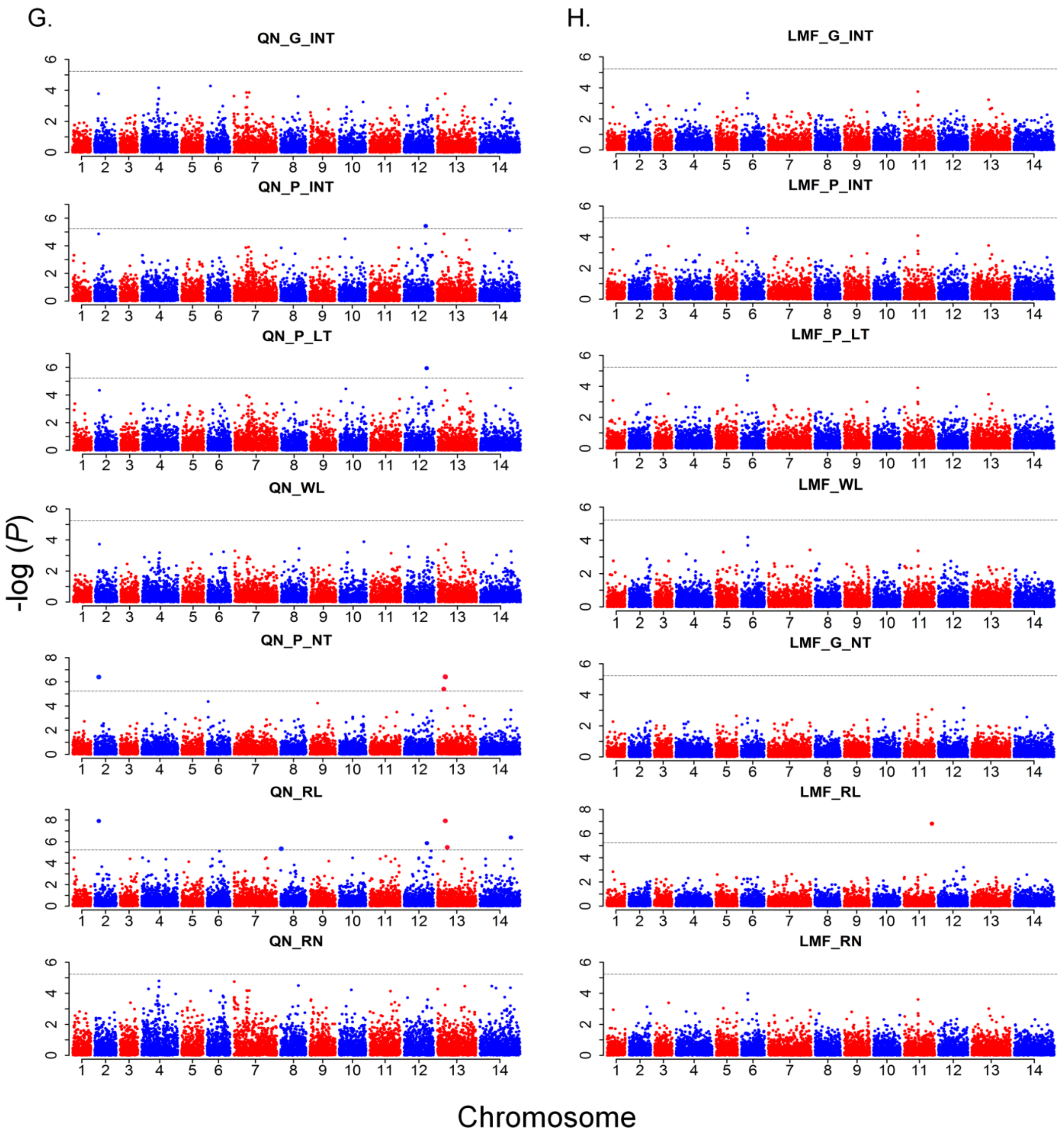
G.
QN_G_INT
QN_P_INT
QN_P_LT
QN_WL
QN_P_NT
QN_RL
QN_RN
H.
LMF_G_INT
LMF_P_INT
LMF_P_LT
LMF_WL
LMF_G_NT
LMF_RL
LMF_RN
-log (P)
Chromosome

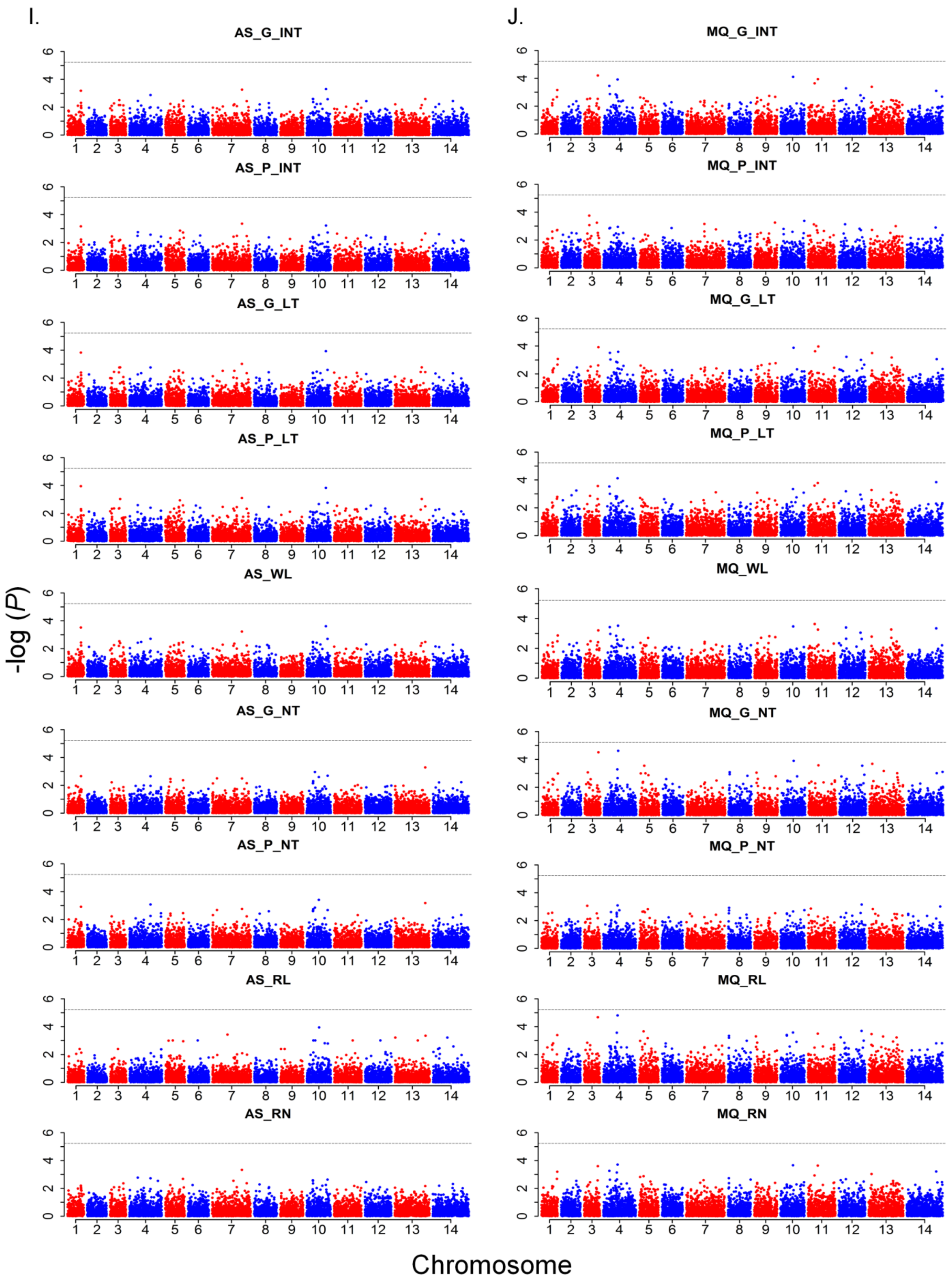
I.
J.
AS_G_INT
AS_P_INT
AS_G_LT
AS_P_LT
AS_WL
AS_G_NT
AS_P_NT
AS_RL
AS_RN
MQ_G_INT
MQ_P_INT
MQ_G_LT
MQ_P_LT
MQ_WL
MQ_G_NT
MQ_P_NT
MQ_RL
MQ_RN
-log (P)
Chromosome

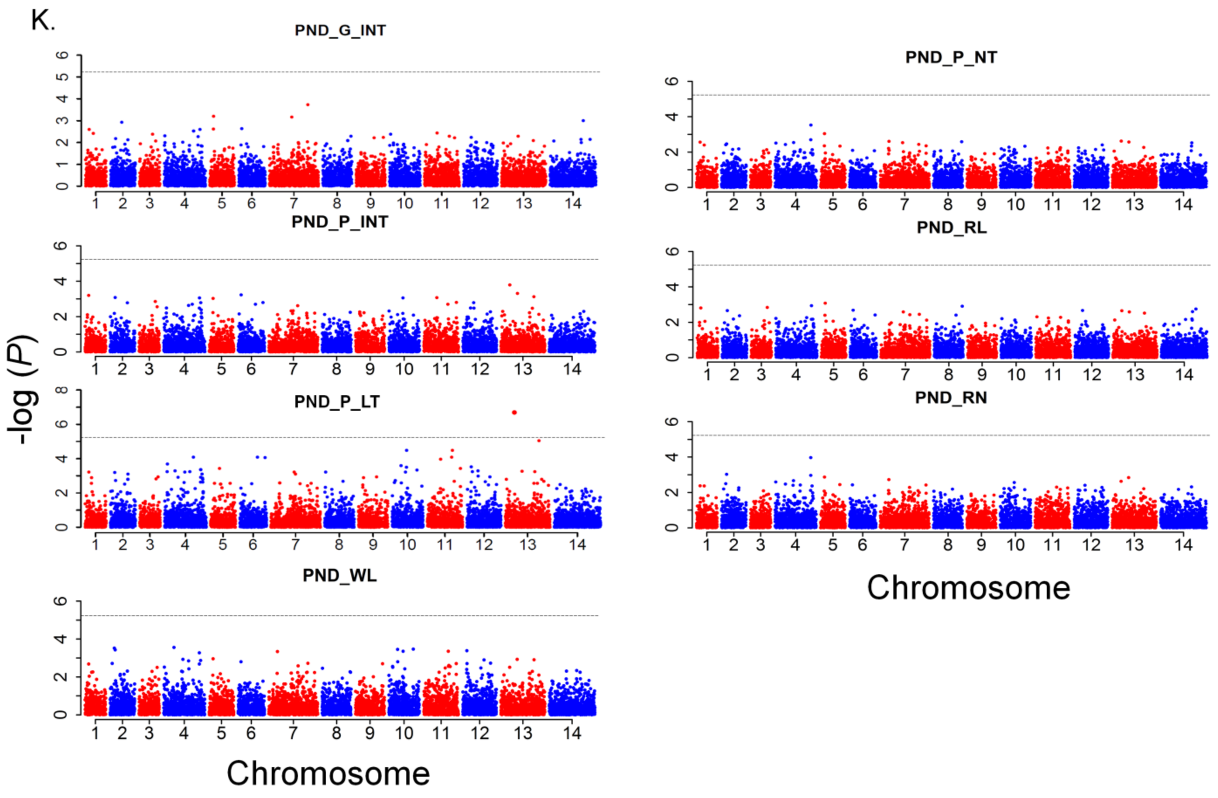

Figure S5. Manhattan plots of the genome-wide association tests. Values of –log(*P*) for 10 drugs were plotted against chromosomal positions. Manhattan plots not shown in Figure 3 are presented, with A for chloroquine (CQ), B for sulfadoxine-pyrimethamine (SP), C for ring-survival rates from ring-stage survival assay, D for dihydroartemisinin (DHA), E for artemether (AM), F for piperaquine (PPQ), G for quinine (QN), H for lumefantrine (LMF), I for artesunate (AS), J for mefloquine (MQ), and K for pyronaridine (PND). Dashed line indicates the threshold for statistical significance. G_LT: GEMMA with log-transformed phenotypes; P_LT: PLINK with log-transformed phenotypes; G_INT: GEMMA with INT-transformed phenotypes; P_INT: Plink with INT-transformed phenotypes; WL: WarpedLMM; G_NT: GEMMA with non-transformed phenotypes; P_NT: PLINK with non-transformed phenotypes; RL: linear regression in R with non-transformed phenotypes; RN: non-parametric regression in R with non-transformed phenotypes.

Table S1. *In vitro* IC50s (nM/μg/ml*) to 10 antimalarial drugs and ring-stage survival rates of 3D7 and 94 culture-adapted clinical isolates.

Drug	Clinical isolates		3D7	P-value (compared with 3D7)
	Median (IQR)	Range	Mean	
RSA	0.7 (0 – 2.2)	0 ~ 64.0	0	0.0003
DHA	1.9 (1.4 – 3.0)	0.5 ~ 6.4	1.2	< 0.0001
AS	2.5 (1.7 – 3.1)	0.6 ~ 7.5	1.7	< 0.0001
AM	2.5 (1.8 – 3.3)	0.8 ~ 9.0	1.9	< 0.0001
LMF	8.6 (4.8 – 13.1)	1.6 ~ 53.9	5.6	< 0.0001
MQ	21.2 (14.9 – 28.7)	7.1 ~ 62.7	13.7	< 0.0001
CQ	1296 (855.5 – 4968.0)	13.5 ~ 4968.0	18.0	< 0.0001
QN	48.0 (32.4 – 76.2)	9.2 ~ 202.5	15.3	< 0.0001
SP	139.7 (92.2 – 178.7)	0.1 ~ 407.3	0.2	< 0.0001
	Mean (95% CI)			
PND	8.1 (7.3 – 8.8)	0.9 ~ 19.8	4.1	< 0.0001
PPQ	22.9 (21.5 – 24.3)	9.5 ~ 40.6	14.7	< 0.0001

Ring-survival rates from ring-stage survival assay (RSA); dihydroartemisinin (DHA); artesunate (AS); artemether (AM); lumefantrine (LMF); mefloquine (MQ); chloroquine (CQ); quinine (QN); sulfadoxine-pyrimethamine (SP); pyronaridine (PND); and piperaquine (PPQ).

Table S2. Genomic loci significantly associated with altered drug sensitivities identified by GWAS. *P*-values of 5.83E-06 or higher are marked in red.

	SNP ID	Chromosome	Position	Gene ID	Function	Normalized					Un-normalized			
						**Log_transformation**		**INT_transformation**			GEMMA	Plink	R_linear	R_non-parametric
						GEMMA	Plink	GEMMA	Plink	WarpedLMM				
**CQ**	Pf_01_000498466	1	497317	PF3D7_0113100	surface-associated interspersed protein 1.1 (SURFIN 1.1) (SURF1.1)	0.000649	2.95E-06	0.07121	0.02501	0.025967	0.087104	0.09164	0.059959	0.0587322
	Pf_01_000515354	1	514205	PF3D7_0113600	surface-associated interspersed protein 1.2 (SURFIN 1.2), pseudogene (SURF1.2)	1.43E-09	6.90E-07	0.00062	0.01192	0.0002665	0.017364	0.04597	0.044849	0.0038003
	Pf_03_000882870	3	886200	null		0.000428	3.73E-07	0.00525	0.00049	0.0056316	0.007961	0.02434	0.018972	0.0009131
	Pf_03_001035406	3	1038736	null		6.63E-08	1.66E-07	0.00023	0.00137	8.33E-05	0.003731	0.009573	0.008182	0.000437
	Pf_04_000745757	4	738775	PF3D7_0416900	conserved Plasmodium protein, unknown function	6.23E-06	6.64E-07	0.0013	0.00144	0.0026641	0.003006	0.0246	0.018711	0.0006241
	Pf_04_000893351	4	886369	PF3D7_0419900	phosphatidylinositol 4-kinase, putative	0.0002261	5.44E-06	2.57E-05	8.99E-05	0.0006545	0.000446	0.007257	0.001986	2.30E-05
	Pf_04_001025113	4	1020673	null		2.47E-05	3.03E-06	0.00016	0.00038	0.0002238	0.000813	0.00418	0.006687	8.47E-05
	Pf_05_000132297	5	132297	PF3D7_0503200	conserved Plasmodium protein, unknown function	0.0021873	4.53E-06	0.03403	0.00734	0.0162238	0.070597	0.06802	0.047272	0.0241096
	Pf_05_001199276	5	1199283	PF3D7_0529400.2	conserved Plasmodium protein, unknown function	7.54E-06	5.60E-07	0.01554	0.01167	0.0114254	0.061657	0.07408	0.225325	0.0196403
	Pf_06_000394837	6	394841	null		5.14E-05	2.16E-07	0.03382	0.00975	0.0200351	0.208865	0.0484	0.506862	0.1116549
	Pf_06_001040345	6	1040344	PF3D7_0625600	poly(A) polymerase PAP, putative	9.56E-09	1.21E-06	0.00192	0.02398	0.000903	0.018539	0.05857	0.23305	0.0043483
	Pf_06_001122480	6	1122479	null		0.0021873	4.53E-06	0.03403	0.00734	0.0162238	0.070597	0.06802	0.047272	0.0241096
	Pf_06_001206228	6	1206227	PF3D7_0629300	phospholipase, putative (PL)	1.69E-09	9.98E-09	0.00037	0.00235	0.0002625	0.006518	0.02395	0.069693	0.0009718
	Pf_06_001215070	6	1215068	PF3D7_0629500	amino acid transporter, putative	4.33E-06	6.72E-08	3.83E-06	1.19E-05	3.65E-05	6.19E-05	0.00194	0.001156	5.25E-06
	Pf_06_001222466	6	1222464	PF3D7_0629700	SET domain protein, putative (SET1)	0.0021873	4.53E-06	0.03403	0.00734	0.0162238	0.070597	0.06802	0.047272	0.0241096
	Pf_06_001224779	6	1224777	PF3D7_0629700	SET domain protein, putative (SET1)	5.56E-07	1.15E-06	0.00053	0.00312	0.0005955	0.009428	0.02606	0.021409	0.0013742
	Pf_06_001296408	6	1296405	null		6.51E-05	1.75E-06	0.00461	0.00221	0.0023485	0.008841	0.01965	0.01553	0.0033984
	Pf_07_000460216	7	null	null		1.90E-15	1.03E-13	8.65E-07	1.17E-05	2.87E-06	0.000187	0.003347	0.003626	1.69E-05
	Pf_07_000459787	7	404407	PF3D7_0709000	**chloroquine resistance transporter (CRT)**	2.70E-16	1.16E-13	1.47E-05	0.00015	9.53E-06	0.001732	0.007353	0.019113	0.000189
	Pf_07_000461218	7	405838	PF3D7_0709000	**chloroquine resistance transporter (CRT)**	1.90E-15	1.03E-13	8.65E-07	1.17E-05	2.87E-06	0.000187	0.003347	0.003626	1.69E-05
	Pf_07_000463814	7	408434	PF3D7_0709100	Cg1 protein	0.0082081	1.68E-06	0.02644	0.00173	0.0187675	0.034046	0.05161	0.020677	0.0068769
	Pf_07_000463819	7	408439	PF3D7_0709100	Cg1 protein	1.41E-05	1.78E-10	0.00203	7.41E-05	0.0008618	0.0041	0.01514	0.011869	0.0004952
	Pf_07_000465512	7	410132	PF3D7_0709100	Cg1 protein	0.0033338	7.87E-07	0.01207	0.00049	0.005385	0.015588	0.03793	0.009809	0.0022307
	Pf_07_000465764	7	410384	PF3D7_0709100	Cg1 protein	1.41E-05	1.78E-10	0.00203	7.41E-05	0.0008618	0.0041	0.01514	0.011869	0.0004952
	Pf_07_000468172	7	412792	PF3D7_0709200	glutaredoxin-like protein (GLP3)	2.70E-16	1.16E-13	1.47E-05	0.00015	9.53E-06	0.001732	0.007353	0.019113	0.000189
	Pf_07_000475935	7	420555	PF3D7_0709300	Cg2 protein (CG2)	1.26E-06	4.63E-10	0.00568	0.00151	0.0026239	0.016682	0.02189	0.042977	0.0062695
	Pf_07_000475948	7	420568	PF3D7_0709300	Cg2 protein (CG2)	1.26E-06	4.63E-10	0.00568	0.00151	0.0026239	0.016682	0.02189	0.042977	0.0062695
	Pf_07_000482133	7	426753	PF3D7_0709400	Cg7 protein	2.63E-05	2.08E-06	1.23E-05	6.00E-05	0.0001252	9.79E-05	0.001515	0.001448	1.25E-05
	Pf_07_000490793	7	435413	PF3D7_0709700	lysophospholipase, putative	2.33E-09	4.14E-07	0.00072	0.01108	0.0006503	0.01897	0.06288	0.238914	0.0035505
	Pf_07_000505396	7	450016	PF3D7_0710000	conserved Plasmodium protein, unknown function	1.22E-06	1.59E-06	0.00017	0.00102	0.0003089	0.003918	0.0109	0.008965	0.0003859
	Pf_07_000505412	7	450032	PF3D7_0710000	conserved Plasmodium protein, unknown function	1.22E-06	1.59E-06	0.00017	0.00102	0.0003089	0.003918	0.0109	0.008965	0.0003859
	Pf_08_001063726	8	1062654	PF3D7_0824400	nucleoside transporter 2 (NT2)	0.006662	1.56E-07	0.0868	0.00025	0.0809946	0.221825	0.02715	0.022067	0.1600467
	Pf_09_001022353	9	1022353	null		0.0021873	4.53E-06	0.03403	0.00734	0.0162238	0.070597	0.06802	0.047272	0.0241096
	Pf_09_001335478	9	1335489	null		2.27E-05	4.60E-08	0.00201	0.00058	0.0008815	0.012719	0.01286	0.007921	0.0020966
	Pf_10_000924465	10	924467	null		8.45E-05	4.88E-06	0.00092	0.00068	0.0011007	0.001153	0.02401	0.012773	0.0002576
	Pf_11_000985769	11	988855	null		0.0021873	4.53E-06	0.03403	0.00734	0.0162238	0.070597	0.06802	0.047272	0.0241096
	Pf_11_001123028	11	1126115	PF3D7_1129100	parasitophorous vacuolar protein 1 (PV1)	3.69E-06	0.000313	0.00813	0.08378	0.0051527	0.043441	0.0948	0.108944	0.0226463

	Pf_11_001291572	11	1294661	PF3D7_1133400	apical membrane antigen 1 (AMA1)	0.0021873	4.53E-06	0.03403	0.00734	0.0162238	0.070597	0.06802	0.047272	0.0241096
	Pf_12_001322488	12	1322498	PF3D7_1231900	conserved Plasmodium protein, unknown function	0.0010754	2.90E-06	0.03548	0.00936	0.0191237	0.04473	0.05396	0.104249	0.0196302
	Pf_12_001527014	12	1527026	null		2.99E-06	0.000131	0.00056	0.00665	0.0004443	0.009653	0.02239	0.02261	0.0010882
	Pf_12_001662982	12	1662994	PF3D7_1239800	conserved Plasmodium protein, unknown function	0.0021873	4.53E-06	0.03403	0.00734	0.0162238	0.070597	0.06802	0.047272	0.0241096
	Pf_14_001023081	14	1022943	null		0.0016957	1.59E-06	0.01432	0.0021	0.0116165	0.024051	0.02927	0.058356	0.0041481
	Pf_14_001068527	14	1068389	PF3D7_1427300	conserved Plasmodium protein, unknown function	0.0021873	4.53E-06	0.03403	0.00734	0.0162238	0.070597	0.06802	0.047272	0.0241096
	Pf_14_001331699	14	1332746	PF3D7_1433500	DNA topoisomerase 2 (TOP2)	0.0021873	4.53E-06	0.03403	0.00734	0.0162238	0.070597	0.06802	0.047272	0.0241096
	Pf_14_001997153	14	1998198	PF3D7_1448600	SNARE protein, putative (VTI1)	7.54E-06	5.60E-07	0.01554	0.01167	0.0114254	0.061657	0.07408	0.225325	0.0196403
	Pf_14_002235181	14	2236232	PF3D7_1454400	aminopeptidase P (APP)	0.0021873	4.53E-06	0.03403	0.00734	0.0162238	0.070597	0.06802	0.047272	0.0241096
	Pf_14_002235182	14	2236233	PF3D7_1454400	aminopeptidase P (APP)	0.0021873	4.53E-06	0.03403	0.00734	0.0162238	0.070597	0.06802	0.047272	0.0241096
	Pf_14_002381042	14	2382094	PF3D7_1457900	conserved Plasmodium protein, unknown function	0.0021873	4.53E-06	0.03403	0.00734	0.0162238	0.070597	0.06802	0.047272	0.0241096
**SP**	Pf_01_000515354	1	514205	PF3D7_0113600	surface-associated interspersed protein 1.2 (SURFIN 1.2), pseudogene (SURF1.2)	3.06E-09	8.45E-11	0.00022	0.00058	0.0001861	0.002226	0.0146	0.007317	0.0043482
	Pf_04_000140295	4	133838	PF3D7_0402200	surface-associated interspersed protein 4.1 (SURFIN 4.1), pseudogene (SURF4.1)	3.12E-05	2.61E-08	0.01334	2.16E-05	0.0352381	0.05067	0.000106	0.177125	0.0226254
	Pf_04_000358945	4	352022	PF3D7_0406800	conserved Plasmodium protein, unknown function	8.19E-06	3.97E-06	8.14E-05	0.00034	0.0001236	0.000125	0.000832	0.002767	0.0002784
	Pf_04_000680587	4	673604	PF3D7_0415200	conserved Plasmodium protein, unknown function	4.98E-06	4.63E-05	6.22E-05	0.00014	0.0003929	0.000173	0.000635	0.000234	0.0009103
	Pf_04_000716495	4	709512	PF3D7_0416200	conserved protein, unknown function	7.69E-06	7.74E-07	0.00629	0.00662	0.0041709	0.020852	0.02566	0.011212	0.0166578
	Pf_04_000745757	4	738775	PF3D7_0416900	conserved Plasmodium protein, unknown function	4.01E-08	0.000114	0.00318	0.00498	0.0058148	0.010496	0.02359	0.042893	0.0105961
	Pf_04_000755243	4	748262	PF3D7_0417200	**bifunctional dihydrofolate reductase-thymidylate synthase (DHFR-TS)**	2.09E-13	1.03E-11	1.40E-06	6.88E-06	1.25E-05	5.13E-06	5.79E-06	0.000176	5.49E-05
	Pf_05_001199276	5	1199283	PF3D7_0529400.2	conserved Plasmodium protein, unknown function	2.69E-06	1.50E-08	0.00305	0.00288	0.0027001	0.015049	0.01699	0.098505	0.0196399
	Pf_06_000411589	6	411593	PF3D7_0609600	hypothetical protein	0.0228156	1.71E-06	0.5524	0.01056	0.7097735	0.886666	0.03988	0.718349	0.7596068
	Pf_06_001040345	6	1040344	PF3D7_0625600	poly(A) polymerase PAP, putative	7.75E-07	5.29E-07	0.00209	0.00657	0.0013641	0.00231	0.01245	0.099098	0.0056644
	Pf_07_000460216	7	460216	null		3.27E-08	7.02E-06	0.0014	0.00292	0.0059795	0.015901	0.047	0.154038	0.0033408
	Pf_07_000461218	7	405838	PF3D7_0709000	chloroquine resistance transporter (CRT)	3.27E-08	7.02E-06	0.0014	0.00292	0.0059795	0.015901	0.047	0.154038	0.0033408
	Pf_07_000490793	7	435413	PF3D7_0709700	lysophospholipase, putative	5.78E-07	1.92E-07	0.00087	0.00209	0.0008668	0.002385	0.01319	0.097888	0.0053053
	Pf_11_001123028	11	1126115	PF3D7_1129100	parasitophorous vacuolar protein 1 (PV1)	7.26E-06	3.58E-06	0.0041	0.01637	0.0046591	0.015392	0.0863	0.047658	0.0330804
	Pf_12_001791802	12	1791815	PF3D7_1242200	queuine tRNA-ribosyltransferase, putative (TGT)	0.0001947	5.61E-06	0.00421	0.00393	0.0027514	0.016102	0.01715	0.084985	0.0143899
	Pf_13_000192968	13	192766	PF3D7_1303800	conserved Plasmodium protein, unknown function	7.33E-05	4.09E-12	0.06709	0.1845	0.0798576	0.204403	0.3982	0.188762	0.4046535
	Pf_13_002814814	13	2837820	PF3D7_1372100	Plasmodium exported protein (PHISTb), unknown function (GEXP04)	0.0084746	7.28E-07	0.00463	0.0019	0.0223984	0.015687	0.008058	0.006529	0.0183202
	Pf_14_001997153	14	1998198	PF3D7_1448600	SNARE protein, putative (VTI1)	2.69E-06	1.50E-08	0.00305	0.00288	0.0027001	0.015049	0.01699	0.098505	0.0196399
**RSA**	Pf_01_000114946	1	114712	PF3D7_0102500	erythrocyte binding antigen-181 (EBA181)	0.0254447	0.009494	1.74E-02	0.00569	0.0024093	1.33E-05	1.21E-06	3.58E-06	0.2416628
	Pf_02_000060099	2	60099	null		0.0051864	0.000551	4.75E-04	1.28E-05	0.0001156	1.18E-08	1.28E-12	1.80E-10	0.1475304
	Pf_02_000062496	2	62496	null		0.0037624	8.98E-05	4.15E-04	4.65E-06	0.0014614	9.50E-06	4.25E-08	2.88E-07	0.0350197
	Pf_02_000233242	2	233242	PF3D7_0205800	conserved Plasmodium protein, unknown function	0.1407366	0.325	5.24E-02	0.1548	0.0062551	1.65E-07	2.08E-05	4.53E-05	0.7928447
	Pf_02_000687213	2	687214	PF3D7_0216600	MtN3-like protein	0.1275845	0.2355	8.97E-03	0.1265	0.2253242	3.83E-10	0.2579	0.220711	0.4594366
	Pf_02_000840796	2	840798	PF3D7_0220800	cytoadherence linked asexual protein 2 (CLAG2)	0.0522741	0.06781	2.82E-02	0.0253	0.0084792	0.000269	4.17E-06	2.13E-05	0.0064921
	Pf_03_000826058	3	829388	PF3D7_0319700	ABC transporter I family member 1, putative (ABCI3)	0.0056297	0.000494	2.11E-03	0.00012	0.001214	3.43E-05	8.83E-07	4.09E-06	0.0571112
	Pf_03_000989455	3	992785	PF3D7_0323700	U4/U6.U5 tri-snRNP-associated protein 1, putative (SART1)	0.3003362	0.2536	1.66E-01	0.1245	0.0004203	5.30E-05	1.75E-05	4.17E-08	0.7928447
	Pf_04_000170193	4	163735	null		0.0824671	0.0878	4.06E-02	0.04782	0.0023103	1.60E-06	1.56E-05	3.26E-05	0.5472846
	Pf_04_000496002	4	489105	PF3D7_0410800	conserved Plasmodium protein, unknown function	0.0122241	0.000419	1.47E-03	1.74E-05	0.0013769	2.06E-08	2.30E-11	3.73E-10	0.1679728
	Pf_04_000496634	4	496634	null		0.0280563	0.002166	6.69E-03	0.00024	0.0045562	7.27E-06	5.04E-08	3.45E-07	0.2154618
	Pf_05_000092998	5	92998	PF3D7_0501800	chromosome assembly factor 1 (CAF1)	0.1125433	0.1504	4.64E-02	0.1011	0.0024133	7.23E-07	1.60E-05	4.73E-05	0.7928447
	Pf_05_000923028	5	923033	PF3D7_0522400	conserved Plasmodium protein, unknown function	0.154214	0.1451	7.68E-02	0.06584	0.0058277	2.47E-05	1.22E-05	2.99E-08	0.8353021
	Pf_05_000952150	5	952155	PF3D7_0522900	zinc finger protein, putative	0.0202155	0.009579	4.19E-03	0.00182	0.0015619	2.56E-06	3.52E-06	1.25E-05	0.2579744

	Pf_06_000103657	6	103660	PF3D7_0602400	elongation factor G (EF-G)	2.39E-05	2.19E-07	2.75E-07	7.66E-10	1.59E-08	5.85E-18	3.67E-21	1.97E-18	0.0136651
	Pf_06_000105827	6	105830	PF3D7_0602500	geranylgeranyltransferase, putative	2.39E-05	2.19E-07	2.75E-07	7.66E-10	1.59E-08	5.85E-18	3.67E-21	1.97E-18	0.0136651
	Pf_06_000232367	6	232370	PF3D7_0605600	nucleoside diphosphate kinase, putative	0.0674311	0.04506	2.63E-02	0.01526	0.0004203	3.38E-08	1.17E-08	5.86E-21	0.6408357
	Pf_06_001300044	6	1300041	null		0.3034478	0.253	1.67E-01	0.1242	0.025694	5.38E-05	1.72E-05	4.35E-08	0.7928447
	Pf_07_001156076	7	1100698	PF3D7_0726200	serine/threonine protein kinase, FIKK family (FIKK7.1)	0.0051873	0.000425	4.75E-04	1.66E-05	0.0003735	4.90E-09	1.98E-11	3.70E-10	0.1679728
	Pf_07_001259185	7	1203807	PF3D7_0728100	conserved Plasmodium membrane protein, unknown function	0.0298588	0.001085	7.49E-03	0.00011	0.0170558	0.000265	1.55E-06	9.28E-06	0.1544782
	Pf_08_000406789	8	405667	PF3D7_0808000	conserved Plasmodium protein, unknown function	0.002695	0.00312	8.09E-04	0.00039	0.0012972	0.000489	3.26E-05	1.33E-06	9.98E-05
	Pf_08_001042265	8	1041193	PF3D7_0823600	lipoate-protein ligase B (LipB)	0.0462277	0.03363	2.47E-02	0.01923	0.0004387	7.46E-07	9.40E-06	2.43E-05	0.2738481
	Pf_08_001175091	8	1174019	PF3D7_0827100	translation initiation factor IF-2, putative (IF2c)	0.00503	0.001472	3.02E-03	0.00085	1.86E-05	2.31E-08	2.16E-09	9.03E-09	0.0516428
	Pf_09_000428857	9	428865	null		0.0020052	6.04E-05	1.79E-04	2.22E-06	5.34E-05	1.12E-08	1.33E-11	2.60E-10	0.0583523
	Pf_09_000510564	9	510573	null		0.0028107	7.47E-05	2.97E-04	3.65E-06	0.0009807	1.30E-05	1.08E-07	6.85E-07	0.0439845
	Pf_09_001155688	9	1155698	null		0.0138072	0.003789	2.83E-03	0.00054	1.84E-06	2.08E-06	9.61E-08	4.25E-07	0.1928651
	Pf_10_000260410	10	260411	null		0.1049032	0.02123	7.40E-02	0.00893	0.0076738	0.000133	5.75E-07	1.12E-05	0.2649287
	Pf_10_000721204	10	721205	PF3D7_1018100	conserved Plasmodium protein, unknown function	0.1752327	0.04519	1.17E-01	0.02378	0.011312	0.000285	2.77E-06	1.97E-05	0.355356
	Pf_10_001207823	10	1206581	PF3D7_1029600	adenosine deaminase (ADA)	0.0824671	0.0878	4.06E-02	0.04782	0.0023103	1.60E-06	1.56E-05	3.26E-05	0.5472846
	Pf_11_000271688	11	274072	PF3D7_1106500	conserved Plasmodium protein, unknown function	0.000139	1.52E-06	4.91E-05	3.77E-07	1.22E-05	3.01E-07	1.44E-09	1.37E-08	0.0036591
	Pf_11_001191501	11	1194588	PF3D7_1131000	RNA-binding protein s1, putative	0.0280366	0.00086	1.45E-02	0.03418	0.0005072	1.59E-07	8.46E-05	0.000422	0.3964249
	Pf_11_001203353	11	1206440	PF3D7_1131400	conserved Plasmodium protein, unknown function	2.39E-05	2.19E-07	2.75E-07	7.66E-10	1.59E-08	5.85E-18	3.67E-21	1.97E-18	0.0136651
	Pf_12_000223536	12	223538	PF3D7_1205100	O-phosphoseryl-tRNA(Sec) selenium transferase, putative (SEPSECS)	0.0002775	3.19E-06	1.20E-04	7.73E-07	1.73E-05	8.41E-07	2.03E-09	2.23E-08	0.0070067
	Pf_12_002217999	12	2218016	null		0.0002011	0.000132	1.88E-06	1.27E-05	2.23E-05	3.23E-15	1.16E-09	1.56E-09	0.0150638
	Pf_13_000426481	13	426280	PF3D7_1309200	protein phosphatase PPM6, putative (PPM6)	0.1407366	0.325	5.24E-02	0.1548	0.0062551	1.65E-07	2.08E-05	4.53E-05	0.7928447
	Pf_13_001191586	13	1191385	PF3D7_1328200	conserved Plasmodium protein, unknown function	0.0084009	0.000357	9.01E-04	1.55E-05	0.0008616	1.29E-08	2.06E-11	3.68E-10	0.1679728
	Pf_13_001229879	13	1229678	PF3D7_1329100	myosin C (MyoC)	0.0334969	0.001066	3.76E-03	4.50E-05	0.0486997	5.85E-05	8.16E-08	5.43E-07	0.1956603
	Pf_13_001231344	13	1231143	PF3D7_1329100	myosin C (MyoC)	0.0041246	0.000841	1.28E-04	1.67E-05	0.0041779	1.86E-06	3.19E-07	2.19E-06	0.2174127
	Pf_13_001718319	13	1718288	PF3D7_1343400	**DNA repair protein RAD5, putative (RAD5)**	0.0954203	0.05452	3.91E-02	0.01785	0.0040211	1.83E-07	1.38E-08	4.70E-08	0.6408357
	Pf_14_000443122	14	443123	PF3D7_1411000.2	conserved Plasmodium protein, unknown function	0.0219934	0.00371	1.14E-02	0.00193	0.0001557	3.31E-07	4.32E-09	1.57E-08	0.0831262
	Pf_14_000959304	14	959160	PF3D7_1423700	conserved Plasmodium protein, unknown function	0.0174946	0.004404	2.29E-03	0.00039	0.0016651	2.57E-07	4.05E-08	3.95E-07	0.2945838
	Pf_14_001608681	14	1609726	PF3D7_1439500	CCAAT-binding transcription factor, putative	0.0824671	0.0878	4.06E-02	0.04782	0.0023103	1.60E-06	1.56E-05	3.26E-05	0.5472846
	Pf_14_002195648	14	2196699	PF3D7_1453500	pyridine nucleotide transhydrogenase, putative	0.0373039	0.01662	6.30E-03	0.00182	0.0054348	2.00E-06	9.63E-08	4.31E-07	0.5232135
	Pf_14_002409641	14	2410693	null		0.0374451	0.02448	1.22E-02	0.00665	0.0003644	8.58E-08	9.95E-09	3.70E-08	0.3239471
	Pf_14_002747615	14	2748665	PF3D7_1467200	WD repeat-containing protein 79, putative (WDR79)	0.1515938	0.04989	6.94E-02	0.01658	0.007754	6.66E-07	1.32E-08	4.71E-08	0.6408357
**DHA**	Pf_07_000152148	7	null	null		0.0004784	0.01748	0.00046	0.025	0.0010324	4.82E-06	0.01964	0.02564	0.0008028
	Pf_08_001020335	8	1019263	PF3D7_0823100	RWD domain-containing protein, putative	1.49E-05	0.002164	2.67E-05	0.00433	0.000244	3.71E-07	0.002002	0.002626	0.0001568
	Pf_10_000490647	10	490648	PF3D7_1012700	**NLI interacting factor-like phosphatase, putative (NIF4)**	7.78E-07	4.72E-05	3.99E-06	0.0001	1.92E-05	2.44E-06	7.02E-05	6.57E-05	4.48E-06
	Pf_10_000490719	10	490720	PF3D7_1012700	**NLI interacting factor-like phosphatase, putative (NIF4)**	7.78E-07	4.72E-05	3.99E-06	0.0001	1.92E-05	2.44E-06	7.02E-05	6.57E-05	4.48E-06
	Pf_10_000497460	10	497461	PF3D7_1012900	**autophagy-related protein 18, putative (ATG18)**	1.16E-06	7.47E-05	5.80E-06	0.00016	2.94E-05	2.73E-06	8.62E-05	8.34E-05	7.02E-06
**AM**	Pf_04_000981058	4	976619	null		4.69E-06	3.10E-05	3.26E-06	2.24E-05	2.32E-05	0.000169	0.00083	0.001038	1.04E-05
	Pf_08_000295942	8	294826	PF3D7_0805300	conserved Plasmodium protein, unknown function	8.49E-05	0.002768	0.00016	0.0022	0.0004141	2.90E-06	0.003039	0.001564	0.0001424
	Pf_10_000490647	10	490648	PF3D7_1012700	**NLI interacting factor-like phosphatase, putative (NIF4)**	2.08E-06	6.35E-05	9.97E-07	3.21E-05	1.70E-05	1.78E-05	0.000654	0.000649	2.33E-06
	Pf_10_000490719	10	490720	PF3D7_1012700	**NLI interacting factor-like phosphatase, putative (NIF4)**	2.08E-06	6.35E-05	9.97E-07	3.21E-05	1.70E-05	1.78E-05	0.000654	0.000649	2.33E-06
	Pf_10_000497460	10	497461	PF3D7_1012900	**autophagy-related protein 18, putative (ATG18)**	5.07E-06	0.000125	2.60E-06	7.46E-05	3.81E-05	2.78E-05	0.000981	0.000977	5.78E-06
**PPQ**	Pf_08_000281296	8	280180	PF3D7_0804900	GTPase-activating protein, putative	5.70E-05	0.000697	2.27E-05	0.00031	2.29E-05	9.57E-07	0.000153	9.35E-05	2.38E-05

	Pf_11_000077735	11	77735	null		0.0019733	0.0006	0.00066	0.00013	0.0004125	0.000184	1.29E-06	1.40E-05	0.0041475
	Pf_12_001959796	12	1959796	null		0.0037829	0.000876	0.00153	0.00021	0.0020434	0.000631	3.41E-06	4.03E-05	0.0030263
**QN**	Pf_02_000233242	2	233242	PF3D7_0205800	conserved Plasmodium protein, unknown function	0.0004848	4.65E-05	0.00017	1.40E-05	0.0001877	4.61E-08	4.24E-07	1.22E-08	0.0040659
	Pf_08_001411330	8	8211	null		0.0590991	0.00043	0.03529	0.00014	0.0188749	0.00078	0.001576	4.59E-06	0.1770145
	Pf_12_001776215	12	1776215	null		0.0010993	1.14E-06	0.00196	3.72E-06	0.0013342	9.50E-05	0.000893	1.39E-06	0.0008522
	Pf_13_000263441	13	263239	PF3D7_1305300	conserved Plasmodium protein, unknown function	0.0026675	0.000581	0.00168	0.00029	0.0015047	6.03E-05	3.86E-06	6.77E-05	0.0088545
	Pf_13_000426481	13	426280	PF3D7_1309200	protein phosphatase PPM6, putative (PPM6)	0.0004848	4.65E-05	0.00017	1.40E-05	0.0001877	4.61E-08	4.24E-07	1.22E-08	0.0040659
	Pf_13_000565177	13	564975	PF3D7_1313100	conserved Plasmodium protein, unknown function	0.0020639	0.000255	0.00389	0.00057	0.0057597	1.50E-05	0.000151	3.57E-06	0.0064491
	Pf_14_002756764	14	2757814	PF3D7_1467600	conserved Plasmodium protein, unknown function	0.0134117	3.16E-05	0.007	8.07E-06	0.0029242	0.000125	0.000214	4.14E-07	0.0574322
**LMF**	Pf_11_001938806	11	1941896	PF3D7_1148800	Plasmodium exported protein (hyp11), unknown function	0.1257743	0.03974	0.12426	0.04617	0.1691439	0.000892	3.88E-06	1.52E-07	0.4067803
**PND**	Pf_10_000557656	10	557657	PF3D7_1014100	conserved Plasmodium protein, unknown function	3.97E-06	0.000258	0.00058	0.00772	0.0003569	0.00534	0.005341	0.03503	0.006045
	Pf_10_000993732	10	992490	PF3D7_1023700	conserved Plasmodium protein, unknown function	6.75E-07	3.38E-05	0.00102	0.01366	0.0004409	0.00768	0.007679	0.050884	0.0170785
	Pf_11_001490677	11	1493767	PF3D7_1138000	conserved Plasmodium protein, unknown function	6.75E-07	3.38E-05	0.00102	0.01366	0.0004409	0.00768	0.007679	0.050884	0.0170785
	Pf_12_000107560	12	107560	PF3D7_1201900	conserved protein, unknown function	4.84E-06	0.000306	0.00083	0.0106	0.0004151	0.00575	0.005752	0.036861	0.0073296
	Pf_13_000410057	13	409854	PF3D7_1308900	mRNA-decapping enzyme 2, putative (DCP2)	6.78E-07	2.03E-07	0.00023	0.00016	0.0031113	0.0024	0.002396	0.002233	0.0023724

Chloroquine (CQ); sulfadoxine-pyrimethamine (SP); ring-survival rates from ring-stage survival assay (RSA); dihydroartemisinin (DHA); artemether (AM); piperaquine (PPQ);quinine (QN), lumefantrine (LMF); and pyronaridine (PND).

Table S3. Pairwise LD of SNPs at the *pfcrt*, *dhfr*, *atg18* and *nif4* genomic loci (R^2 > 0.3).

	SNP	Linked SNP	Chr.	Gene of linked SNP	Gene description	Distance (bp)	R^2
CRT	Pf_07_000458717	Pf_07_000459787	7	PF3D7_0709000	chloroquine resistance transporter (CRT)	1070	0.333827
	Pf_07_000458717	Pf_07_000461218	7	PF3D7_0709000	chloroquine resistance transporter (CRT)	2501	0.698413
	Pf_07_000458717	Pf_07_000463814	7	PF3D7_0709100	Cg1 protein	5097	0.586957
	Pf_07_000458717	Pf_07_000463819	7	PF3D7_0709100	Cg1 protein	5102	0.622346
	Pf_07_000458717	Pf_07_000464077	7	PF3D7_0709100	Cg1 protein	5360	0.824074
	Pf_07_000458717	Pf_07_000464123	7	PF3D7_0709100	Cg1 protein	5406	0.824074
	Pf_07_000458717	Pf_07_000465512	7	PF3D7_0709100	Cg1 protein	6795	0.791209
	Pf_07_000458717	Pf_07_000465764	7	PF3D7_0709100	Cg1 protein	7047	0.622346
	Pf_07_000458717	Pf_07_000468172	7	PF3D7_0709200	glutaredoxin-like protein (GLP3)	9455	0.333827
	Pf_07_000458717	Pf_07_000471155	7	PF3D7_0709300	Cg2 protein (CG2)	12438	0.586957
	Pf_07_000458717	Pf_07_000489545	7	null		30828	0.586957
	Pf_07_000459787	Pf_07_000282699	7	PF3D7_0704600	E3 ubiquitin-protein ligase (UT)	177090	0.333827
	Pf_07_000459787	Pf_07_000434049	7	PF3D7_0708300	EKC/KEOPS complex subunit BUD32 (BUD32)	25738	0.387097
	Pf_07_000459787	Pf_07_000458717	7	PF3D7_0709000	chloroquine resistance transporter (CRT)	1070	0.333827
	Pf_07_000459787	Pf_07_000461218	7	PF3D7_0709000	chloroquine resistance transporter (CRT)	1431	0.698413
	Pf_07_000459787	Pf_07_000463819	7	PF3D7_0709100	Cg1 protein	4032	0.333827
	Pf_07_000459787	Pf_07_000465764	7	PF3D7_0709100	Cg1 protein	5977	0.333827
	Pf_07_000459787	Pf_07_000468172	7	PF3D7_0709200	glutaredoxin-like protein (GLP3)	8385	1
	Pf_07_000459787	Pf_07_000471155	7	PF3D7_0709300	Cg2 protein (CG2)	11368	0.586957
	Pf_07_000459787	Pf_07_000472854	7	PF3D7_0709300	Cg2 protein (CG2)	13067	0.387097
	Pf_07_000459787	Pf_07_000475935	7	PF3D7_0709300	Cg2 protein (CG2)	16148	0.622346
	Pf_07_000459787	Pf_07_000475948	7	PF3D7_0709300	Cg2 protein (CG2)	16161	0.622346
	Pf_07_000459787	Pf_07_000489545	7	null		29758	0.586957
	Pf_07_000459787	Pf_07_000490793	7	PF3D7_0709700	lysophospholipase, putative	31006	0.586957
	Pf_07_000459787	Pf_07_000505396	7	PF3D7_0710000	conserved Plasmodium protein, unknown function	45609	0.509779
	Pf_07_000459787	Pf_07_000505412	7	PF3D7_0710000	conserved Plasmodium protein, unknown function	45625	0.509779

	Pf_07_000461218	Pf_07_000458717	7	PF3D7_0709000	chloroquine resistance transporter (CRT)	2501	0.698413
	Pf_07_000461218	Pf_07_000459787	7	PF3D7_0709000	chloroquine resistance transporter (CRT)	1431	0.698413
	Pf_07_000461218	Pf_07_000463814	7	PF3D7_0709100	Cg1 protein	2596	0.409938
	Pf_07_000461218	Pf_07_000463819	7	PF3D7_0709100	Cg1 protein	2601	0.698413
	Pf_07_000461218	Pf_07_000464077	7	PF3D7_0709100	Cg1 protein	2859	0.569972
	Pf_07_000461218	Pf_07_000464123	7	PF3D7_0709100	Cg1 protein	2905	0.569972
	Pf_07_000461218	Pf_07_000465512	7	PF3D7_0709100	Cg1 protein	4294	0.55259
	Pf_07_000461218	Pf_07_000465764	7	PF3D7_0709100	Cg1 protein	4546	0.698413
	Pf_07_000461218	Pf_07_000468172	7	PF3D7_0709200	glutaredoxin-like protein (GLP3)	6954	0.698413
	Pf_07_000461218	Pf_07_000471155	7	PF3D7_0709300	Cg2 protein (CG2)	9937	0.409938
	Pf_07_000461218	Pf_07_000475935	7	PF3D7_0709300	Cg2 protein (CG2)	14717	0.429383
	Pf_07_000461218	Pf_07_000475948	7	PF3D7_0709300	Cg2 protein (CG2)	14730	0.429383
	Pf_07_000461218	Pf_07_000482133	7	PF3D7_0709400	Cg7 protein	20915	0.364973
	Pf_07_000461218	Pf_07_000489545	7	null		28327	0.409938
	Pf_07_000461218	Pf_07_000490793	7	PF3D7_0709700	lysophospholipase, putative	29575	0.409938
	Pf_07_000461218	Pf_07_000505396	7	PF3D7_0710000	conserved Plasmodium protein, unknown function	44178	0.347305
	Pf_07_000461218	Pf_07_000505412	7	PF3D7_0710000	conserved Plasmodium protein, unknown function	44194	0.347305
DHFR	Pf_04_000755243	Pf_04_000680525	4	PF3D7_0415200	conserved Plasmodium protein, unknown function	74720	0.405849
	Pf_04_000755243	Pf_04_000680587	4	PF3D7_0415200	conserved Plasmodium protein, unknown function	74658	0.460372
	Pf_04_000755243	Pf_04_000680618	4	PF3D7_0415200	conserved Plasmodium protein, unknown function	74627	0.405849
	Pf_04_000755243	Pf_04_000745757	4	PF3D7_0416900	conserved Plasmodium protein, unknown function	9487	0.454023
	Pf_04_000755243	Pf_04_000765918	4	PF3D7_0417400	conserved Plasmodium protein, unknown function	10675	0.604167
	Pf_04_000755243	Pf_04_000773649	4	PF3D7_0417400	conserved Plasmodium protein, unknown function	18406	0.301481
	Pf_04_000755220	Pf_04_000745757	4	PF3D7_0416900	conserved Plasmodium protein, unknown function	9464	0.363366
ATG18	Pf_10_000497460	Pf_10_000490647	10	PF3D7_1012700	NLI interacting factor-like phosphatase, putative (NIF4)	6813	0.957871
	Pf_10_000497460	Pf_10_000490719	10	PF3D7_1012700	NLI interacting factor-like phosphatase, putative (NIF4)	6741	0.957871
	Pf_10_000497460	Pf_10_000497631	10	null		171	0.335664
	Pf_10_000497460	Pf_10_000515420	10	null		17960	0.464789
	Pf_10_000497460	Pf_10_000528414	10	PF3D7_1013500	phosphoinositide-specific phospholipase C (PI-PLC)	30954	0.351869

NIF4	Pf_10_000487744	Pf_10_000067421	10	null		420325	0.315377
	Pf_10_000487744	Pf_10_000382041	10	PF3D7_1009400	zinc finger protein, putative	105704	0.428724
	Pf_10_000487744	Pf_10_000501814	10	PF3D7_1013000	zinc finger protein, putative	14070	0.489247
	Pf_10_000487744	Pf_10_001393106	10	PF3D7_1035100	probable protein, unknown function	904120	0.335664
	Pf_10_000487744	Pf_10_001393184	10	PF3D7_1035100	probable protein, unknown function	904198	0.373626
	Pf_10_000487744	Pf_10_001393214	10	PF3D7_1035100	probable protein, unknown function	904228	0.373626
	Pf_10_000487744	Pf_10_001393299	10	PF3D7_1035100	probable protein, unknown function	904313	0.304029
	Pf_10_000490647	Pf_10_000490719	10	PF3D7_1012700	NLI interacting factor-like phosphatase, putative (NIF4)	72	1
	Pf_10_000490647	Pf_10_000497460	10	PF3D7_1012900	autophagy-related protein 18, putative (ATG18)	6813	0.957871
	Pf_10_000490647	Pf_10_000515420	10	null		24773	0.445208
	Pf_10_000490647	Pf_10_000528414	10	PF3D7_1013500	phosphoinositide-specific phospholipase C (PI-PLC)	37767	0.335983
	Pf_10_000490719	Pf_10_000490647	10	PF3D7_1012700	NLI interacting factor-like phosphatase, putative (NIF4)	72	1
	Pf_10_000490719	Pf_10_000497460	10	autophagy-related protein 18, putative (ATG18)		6741	0.957871
	Pf_10_000490719	Pf_10_000515420	10	null		24701	0.445208
	Pf_10_000490719	Pf_10_000528414	10	PF3D7_1013500	phosphoinositide-specific phospholipase C (PI-PLC)	37695	0.335983

Table S4. SNPs/genes under significant positive selection detected using the integrated haplotype scores (iHS).

SNP ID	Chromosome	Position	Gene ID	Function	iHS score
Pf_01_000180370	1	180135	PF3D7_0104100	conserved Plasmodium membrane protein, unknown function	3.527543
Pf_01_000497255	1	496106	PF3D7_0113100	surface-associated interspersed protein 1.1 (SURFIN 1.1) (SURF1.1)	3.296461
Pf_01_000531530	1	529808	PF3D7_0113800	DBL containing protein, unknown function	4.520615
Pf_01_000532101	1	530379	PF3D7_0113800	DBL containing protein, unknown function	3.811156
Pf_01_000533657	1	531935	PF3D7_0113800	DBL containing protein, unknown function	3.211105
Pf_01_000534040	1	532318	PF3D7_0113800	DBL containing protein, unknown function	3.996298
Pf_01_000534087	1	532365	PF3D7_0113800	DBL containing protein, unknown function	4.375165
Pf_02_000843218	2	843220	PF3D7_0220800	cytoadherence linked asexual protein 2 (CLAG2)	3.800708
Pf_02_000845981	2	845983	PF3D7_0220900	Plasmodium exported protein, unknown function, pseudogene	4.529737
Pf_04_000138448	4	131991	PF3D7_0402200	surface-associated interspersed protein 4.1 (SURFIN 4.1), pseudogene (SURF4.1)	3.543212
Pf_04_000613216	4	606233	null		3.499439
Pf_04_000615546	4	608563	null		3.662333
Pf_04_000615710	4	608727	null		3.353782
Pf_04_000618564	4	611581	null		3.636223
Pf_06_001282694	6	1282691	PF3D7_0630600	conserved Plasmodium protein, unknown function	3.198067
Pf_06_001282751	6	1282748	PF3D7_0630600	conserved Plasmodium protein, unknown function	3.838096
Pf_07_000135341	7	79963	PF3D7_0701900	Plasmodium exported protein, unknown function	3.609911
Pf_07_000135438	7	80060	PF3D7_0701900	Plasmodium exported protein, unknown function	3.887396
Pf_07_000135442	7	80064	PF3D7_0701900	Plasmodium exported protein, unknown function	4.06929
Pf_07_000135470	7	80092	PF3D7_0701900	Plasmodium exported protein, unknown function	4.588225
Pf_07_000282506	7	227124	PF3D7_0704600	E3 ubiquitin-protein ligase (UT)	3.642335
Pf_07_000490748	7	435368	PF3D7_0709700	lysophospholipase, putative	3.209422
Pf_07_000520888	7	465508	PF3D7_0710200	conserved Plasmodium protein, unknown function	3.781192
Pf_07_000521308	7	465928	PF3D7_0710200	conserved Plasmodium protein, unknown function	3.273884
Pf_07_000521489	7	466109	PF3D7_0710200	conserved Plasmodium protein, unknown function	3.59323
Pf_07_000521504	7	466124	PF3D7_0710200	conserved Plasmodium protein, unknown function	3.53421
Pf_07_000521513	7	466133	PF3D7_0710200	conserved Plasmodium protein, unknown function	3.53421
Pf_07_000522585	7	467205	PF3D7_0710200	conserved Plasmodium protein, unknown function	4.006322

Pf_07_001209074	7	1153696	PF3D7_0727100	conserved Plasmodium protein, unknown function	3.254946
Pf_07_001227375	7	1171997	PF3D7_0727700	conserved Plasmodium protein, unknown function	3.234425
Pf_07_001414774	7	1359396	PF3D7_0731500	erythrocyte binding antigen-175 (EBA175)	3.428318
Pf_09_001202639	9	1202649	PF3D7_0930300	merozoite surface protein 1 (MSP1)	4.394842
Pf_09_001203325	9	1203335	PF3D7_0930300	merozoite surface protein 1 (MSP1)	3.820165
Pf_09_001203794	9	1203804	PF3D7_0930300	merozoite surface protein 1 (MSP1)	3.632173
Pf_09_001203802	9	1203812	PF3D7_0930300	merozoite surface protein 1 (MSP1)	3.632173
Pf_10_000223013	10	223013	PF3D7_1004800	ADP/ATP carrier protein, putative	3.457744
Pf_10_000223045	10	223045	PF3D7_1004800	ADP/ATP carrier protein, putative	3.795835
Pf_10_000223046	10	223046	PF3D7_1004800	ADP/ATP carrier protein, putative	4.639221
Pf_10_000223064	10	223064	PF3D7_1004800	ADP/ATP carrier protein, putative	3.384442
Pf_10_001319951	10	1318709	PF3D7_1032900	RNA polymerase II-associated protein 1, putative	3.873648
Pf_10_001395188	10	1393947	null		3.685084
Pf_10_001395376	10	1394135	null		3.833066
Pf_11_001016247	11	1019333	PF3D7_1126100	autophagy-related protein 7, putative (ATG7)	3.327825
Pf_11_001124030	11	1127117	PF3D7_1129100	parasitophorous vacuolar protein 1 (PV1)	3.425138
Pf_11_001291289	11	1294378	PF3D7_1133400	apical membrane antigen 1 (AMA1)	3.292095
Pf_11_001292108	11	1295197	PF3D7_1133400	apical membrane antigen 1 (AMA1)	3.159995
Pf_11_001292220	11	1295309	PF3D7_1133400	apical membrane antigen 1 (AMA1)	3.526695
Pf_11_001292244	11	1295333	PF3D7_1133400	apical membrane antigen 1 (AMA1)	3.586375
Pf_12_000059595	12	59595	null		3.17844
Pf_12_002267889	12	2267906	null		4.118928
Pf_13_000106419	13	106316	PF3D7_1301800	surface-associated interspersed protein 13.1 (SURFIN 13.1), pseudogene (SURF13.1)	3.69068
Pf_13_001465883	13	1465685	PF3D7_1335900	sporozoite surface protein 2 (TRAP)	3.203684
Pf_13_001466450	13	1466252	PF3D7_1335900	sporozoite surface protein 2 (TRAP)	3.401529
Pf_14_002613103	14	2614155	PF3D7_1464500	conserved Plasmodium membrane protein, unknown function	3.257594
Pf_14_003120498	14	3121428	PF3D7_1475800	conserved Plasmodium protein, unknown function	4.395597
Pf_14_003120584	14	3121514	PF3D7_1475800	conserved Plasmodium protein, unknown function	5.590858
Pf_14_003120594	14	3121524	PF3D7_1475800	conserved Plasmodium protein, unknown function	4.327432
Pf_14_003193574	14	3194504	PF3D7_1477600	surface-associated interspersed protein 14.1 (SURFIN 14.1) (SURF14.1)	3.213346

SNPs with an integrated haplotype score (iHS) of 3.14 (top 1%) or higher were presented. Known antigens were highlighted in grey.
